# Altitudinal gradients, biogeographic history and microhabitat adaptation affect fine-scale spatial genetic structure in African and Neotropical populations of an ancient tropical tree species

**DOI:** 10.1371/journal.pone.0182515

**Published:** 2017-08-03

**Authors:** Paloma Torroba-Balmori, Katharina B. Budde, Katrin Heer, Santiago C. González-Martínez, Sanna Olsson, Caroline Scotti-Saintagne, Maxime Casalis, Bonaventure Sonké, Christopher W. Dick, Myriam Heuertz

**Affiliations:** 1 Department of Forest Ecology and Genetics, INIA Forest Research Centre, Madrid, Spain; 2 Sustainable Forest Management Research Institute, University of Valladolid - INIA, Palencia, Spain; 3 UMR BIOGECO, INRA, University of Bordeaux, Cestas, France; 4 Institute of Experimental Ecology, University of Ulm, Ulm, Germany; 5 Conservation Biology and Ecology, University of Marburg, Marburg, Germany; 6 UR Écologie des Forêts Méditerranéennes, INRA, Avignon, France; 7 UMR EcoFoG, INRA, Kourou, French Guiana; 8 Ecole Normale Supérieure, Université de Yaoundé I, Yaoundé, Cameroon; 9 Evolutionary Biology and Ecology, Faculté des Sciences, Université Libre de Bruxelles, Brussels, Belgium; 10 Department of Ecology and Evolutionary Biology, University of Michigan, Ann Arbor, Michigan, United States of America; 11 Smithsonian Tropical Research Institute, Republic of Panama; Chinese Academy of Sciences, CHINA

## Abstract

The analysis of fine-scale spatial genetic structure (FSGS) within populations can provide insights into eco-evolutionary processes. Restricted dispersal and locally occurring genetic drift are the primary causes for FSGS at equilibrium, as described in the isolation by distance (IBD) model. Beyond IBD expectations, spatial, environmental or historical factors can affect FSGS. We examined FSGS in seven African and Neotropical populations of the late-successional rain forest tree *Symphonia globulifera* L. f. (Clusiaceae) to discriminate the influence of drift-dispersal vs. landscape/ecological features and historical processes on FSGS. We used spatial principal component analysis and Bayesian clustering to assess spatial genetic heterogeneity at SSRs and examined its association with plastid DNA and habitat features. African populations (from Cameroon and São Tomé) displayed a stronger FSGS than Neotropical populations at both marker types (mean *Sp* = 0.025 *vs*. *Sp* = 0.008 at SSRs) and had a stronger spatial genetic heterogeneity. All three African populations occurred in pronounced altitudinal gradients, possibly restricting animal-mediated seed dispersal. Cyto-nuclear disequilibria in Cameroonian populations also suggested a legacy of biogeographic history to explain these genetic patterns. Conversely, Neotropical populations exhibited a weaker FSGS, which may reflect more efficient wide-ranging seed dispersal by Neotropical bats and other dispersers. The population from French Guiana displayed an association of plastid haplotypes with two morphotypes characterized by differential habitat preferences. Our results highlight the importance of the microenvironment for eco-evolutionary processes within persistent tropical tree populations.

## Introduction

Fine-scale spatial genetic structure (FSGS), the non-random spatial distribution of genotypes within populations, is shaped by microevolutionary processes such as dispersal, local genetic drift and selection [1]. FSGS studies can inform on mechanisms underlying demographic processes and spatial genetic heterogeneity in populations, providing guidance for sustainable forest management and conservation practises (e.g. [2]). One of the most commonly evaluated patterns in FSGS studies in plants is isolation by distance (IBD, [3,4]). The IBD model predicts that, at drift-dispersal equilibrium, genetic differentiation among individuals is an increasing function of geographic distance due to spatially limited isotropic gene dispersal and local genetic drift [1,3,5,6]. A linear relationship is predicted with distance for 1-dimensional populations or with the logarithm of distance for 2-dimensional populations [1,3,5,6]. As pollen and seed dispersal are usually spatially restricted, the strength of FSGS under IBD assumptions can provide information on the historical gene dispersal distance in the population [1,6]. While useful as the basic expected pattern (null model), the IBD model does not consider other features or processes that can constrain gene flow or generate spatial heterogeneity or discontinuities in allele frequencies.

The strength of FSGS depends primarily on an organism’s life history traits, of which life form and breeding system are the most relevant in plants. Indeed, stronger FSGS is found in herbaceous plants than in trees, as well as in partially or completely selfing than in outcrossing species [1]. Population density has also a very important effect, with stronger FSGS found in low-density populations [1,7]. Further, dispersal vectors matter as they determine the scale and spatial pattern of dispersal. In tropical trees for example, it has been shown that animal-pollination typically results in stronger FSGS than wind-pollination, and that gravity- or rodent-mediated seed dispersal generates stronger FSGS than dispersal by birds or larger animals [8,9].

Beyond IBD expectations, intrinsic and extrinsic factors, often associated with landscape features, determine heterogeneity in FSGS patterns. Topographic features or complex relief can directly hinder genetic connectivity and thereby lead to anisotropic and/or heterogeneous FSGS. This occurs for instance if steep slopes or mountain ridges restrict gene flow, which contributes to genetic differentiation even in species with wide-ranging gene dispersal [10,11]. Habitat features can also influence the behaviour of seed and pollen dispersers, affecting the genetic structure of the plants they disperse [12–15]. For example, Jordano *et al*. [16] showed that small birds tended to disperse *Prunus mahaleb* seeds into covered microhabitats but medium-sized birds and small mammals preferentially deposited seed into open habitats. Genetic heterogeneity can also result from historical processes related to range dynamics, such as secondary contact of previously differentiated gene pools [17,18]. Finally, another factor shaping FSGS is habitat-mediated selection, which can generate adaptive differentiation, i.e. isolation by adaptation also known as isolation by environment [19,20]. Although this process first affects only loci under selection, at later stages it can lead to genome-wide differentiation due to hitchhiking [21].

It is challenging to determine whether a given FSGS pattern reflects spatial autocorrelation due to IBD alone, or whether it contains an additional spatial genetic heterogeneity (SGH, i.e. allele frequency discontinuities or locally co-occurring differentiated gene pools [GPs]) signal due to historical or contemporary processes. This is because spatial autocorrelation (the expected result of IBD) affects the analysis of spatial genetic discontinuities, and *vice versa* [4]. Bayesian clustering methods employed to detect SGH can fail to detect genetic clines when genetic structure is weak, but they can also overestimate the number of genetic clusters due to the influence of IBD [22,23]. Incorporating spatial information into clustering methods can improve their results [23,24]. Conversely, methods that quantify FSGS based on the IBD model at drift-dispersal equilibrium cannot independently assess the effect of IBD when genetic discontinuities are present [1]. Moreover, different combinations of historical and contemporary processes can produce similar FSGS patterns, further complicating the inference [25]. Aware of these issues, some authors have used sequential approaches of genetic cluster detection and IBD assessment (or *vice versa*) to infer population genetic processes [18,26], or relied on non-parametric methods to assess heterogeneous spatial genetic patterns [27,28]. A complementary approach to disentangle the factors contributing to FSGS is to compare the FSGS at biparentally inherited nuclear markers to FSGS at maternally inherited markers. Maternal markers will inform about gene flow due to seed dispersal (e.g. [29,30]) and, due to their lower mutation rates, they can identify signatures of processes at deeper temporal scales than nuclear markers [31].

In this study, we set out to investigate FSGS within populations and discriminate the influence of drift-dispersal vs. landscape/ecological features and historical processes on FSGS in the late-successional rainforest tree *Symphonia globulifera* L. f. (Clusiaceae), a species that can be considered a living fossil with a potential Eocene origin [32–34]. *Symphonia globulifera* occurs in tropical Africa and the Neotropics in a wide variety of environments [35] and both pollen and seed are dispersed by numerous animal species in distinct parts of its range (see Table 1). Different *S*. *globulifera* morphotypes co-occur in some regions and occasionally show evidence of habitat specialization [36,37]. Because *S*. *globulifera* populations persisted through multiple geological time periods [30,38,39], we hypothesized that populations should be close to demographic equilibrium and display IBD due to drift-dispersal processes (e.g. [8]). Given the life history traits of our species, i.e. an essentially outcrossed, animal-pollinated and animal-dispersed tropical tree, we expected the FSGS quantified with the *Sp* statistic to be approximately 0.01–0.02 [1,8], probably with substantial variation among populations with different dispersers. We also hypothesized that FSGS should vary among populations because of idiosyncratic ecological characteristics of populations, e.g. a stronger FSGS should *a priori* be expected in populations with marked topography [10,11] or narrow-ranging dispersal [8,9]. Complex interactions between ecological features and each population’s specific history could lead to SGH. Hence, their effects on FSGS are difficult to predict. We thus chose a discovery-driven approach to characterize FSGS with nuclear and plastid markers in seven *S*. *globulifera* populations from Africa and America, identified the population characteristics associated with particular magnitudes or patterns of FSGS, and interpreted FSGS in the light of specific tests on the available data. We addressed the following specific questions: (i) Is within-population FSGS in *S*. *globulifera* in agreement with expectations based on the species’ life history traits, and to what extent does its strength vary among populations? (ii) Is FSGS in agreement with drift-dispersal equilibrium as predicted by IBD theory or are there within-population discontinuities in allele frequencies (SGH)? (iii) Are there any similarities in the strength and patterns of FSGS in groups of populations, and do they concur with, e.g. similar disperser communities, habitat features or biogeographic history?

**Table 1:** Review of animals reported as seed dispersers or pollinators of *Symphonia globulifera* in Africa or the Neotropics and characteristics of their dispersal range. P, pollinator; sd, seed disperser.

Visitors	Cited genus/species	Function	Country or region	Source	References	Dispersal range	References
**Africa**							
sunbirds	*Cyanomitra*, *Nectarinia*, *Cinnyris*, *Chalcomitra*, *Hedydipna*	P	Central and South Africa	bibliographical compilation	[35,40]	max: 50–100 m (*Chalcomitra amethystina*)	[41]
monkeys	*Cercopithecus lhoesti*	sd (defecation, spitting out, transportation in cheek pouches)	Uganda, Cameroon, Gabon	direct observation, seed traps	[42–46]	range: a few meters– 100m (*Cercopithecus* monkeys, seeds >1cm)	[47]
small ruminants	*Cephalophus monticola*, *Hyemoschus aquaticus*	sd (defecation, regurgitation, predation?)	Gabon	direct observation, stomach content	[42,43,48,49]	no information	
hornbills	Putatively: *Tockus fasciatus*, *Bycanistes fistulator*, *B*. *albotibialis*, *Ceratogymna atrata*	sd (defecation?,regurgitation)	Gabon	direct observation, stomach content	[43,49]	max: >500 m (*Ceratogyma atrata*, *C*. *cylindricus*)	[50]
**Neotropics**							
hummingbirds	*Chlorestes notatus*, *Thalurania furcata*	P	Costa Rica, Brazil, French Guiana	direct observation	[51,52]	max: 1–100 m (depending on the species)	[53,54]
perching birds	*Cacicus cela*, *Dacnis lineata*, *Dacnis cayana*, *Chlorophanes spiza*, *Cyanerpes caeruleus*, *Cyanerpes cyaneus*	P	Brazil	direct observation	[52,55]	no information	
lepidoptera	unidentified	P	Brazil, Costa Rica	direct observation	[52,56]	max: 8–10 m (species pollinating *Cnidoscolus urens* in Costa Rica and *Lindenia rivalis* in Belize)	[53,54]
bees	*Trigona cf*. *branneri*	P	Brazil	direct observation	[52]	mean: 260–590 m in buzz-pollinating bees (*Scaptotrigona*, *Trigona*, *Xylocopa*)	[57]
bats	*Artibeus lituratus*, *Artibeus jamaicensis*, *Artibeus watsoni*	sd (exozoochorous)	Costa Rica, French Guiana	direct observation, seed rain under feeding roosts	[58–60]	max: 100 m (*Artibeus lituratus*), max: 25–400 m (*Artibeus jamaicensis*)	[61,62]
scatter-hoarding rodents	unidentified	sd (exozoochorous)	French Guiana	cited	[8,36]	mean: 5–15 m (rodents)	[63,64]
nocturnal arboreal mammals	unidentified	sd (unknown)	French Guiana	cited	[8]	no information	
monkeys	*Leontopithecus rosalia*	sd (unknown, defecation is possible)	Brazil	direct observation	[65,66]	mean: 105 m	[67]
tapirs	*Tapirus terrestris Tapirus bairdii*	sd (defecation)	French Guiana, Central America	stomach content, bibliographical compilation	[49,68,69]	max: 2 km	[70,71]

## Material and methods

### Species description

*Symphonia globulifera* L. f. (Clusiaceae) are generally tall, hermaphroditic rainforest trees widespread throughout tropical Africa, Central and South America [35]. The species is mostly outcrossing [72–74] although selfing can occur, especially in fragmented areas [59,75]. The genus evolved as early as the Eocene, with fossil pollen dated ~45 Ma in the Niger delta [32,33]. *Symphonia globulifera* is currently the only recognized *Symphonia* species outside Madagascar [35,76]. The species colonized America from Africa through trans-Atlantic dispersal some 18–15 Myr ago and has been widespread in both Africa and the Neotropics for millions of years [33]. *Symphonia globulifera* has persisted locally in many sites throughout the Quaternary glaciations [30,38,39,77]. It occurs today in tropical forests, in a range of precipitation and temperature of 650–2,800 mm and 23–27°C, respectively, and from sea level to 2,600 m altitude (in East Africa [35]). Pollinators and seed dispersers vary in different parts of its range, with notable differences between African and Neotropical populations (Table 1). Different morphotypes or suspected ecotypes of *S*. *globulifera* occur in several regions, e.g. a small tree form in Costa Rica [38], a suspected swamp ecotype in West Africa [30]. In Paracou, French Guiana, the common flood-tolerant *S*. *globulifera* morphotype co-occurrs with a *terra firme* morphotype with smaller flowers, smooth bark and adventitious roots but no pneumatophores [37].

### Study sites and plant material

**Fig 1:**
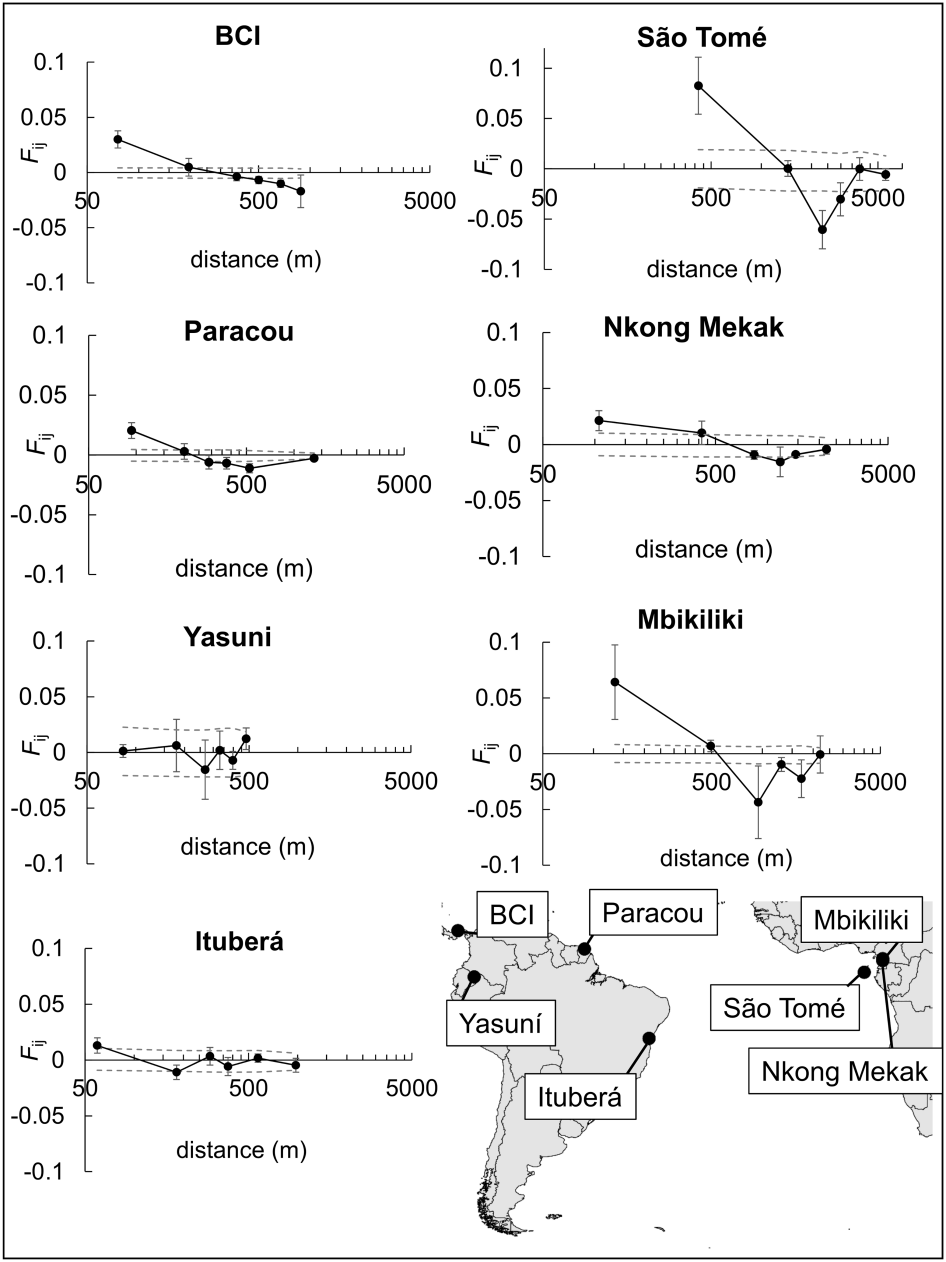
Location of *Symphonia globulifera* populations examined in this paper and kinship-distance relationships within populations. The mean jackknife estimate of the kinship coefficient F_ij_ (± standard error) is plotted per distance class, as well as the permutation-based 95% CI for absence of FSGS (dashed grey lines).

In our study, we examined seven populations from Africa and the Neotropics, all located in mature forests (Fig 1, Table 2). Between 2007 and 2010, we collected leaf or cambium samples on 34–148 georeferenced trees per population and dried samples on silica gel. Sampled trees generally had ≥ 10 cm diameter at breast height (dbh), except in Barro Colorado Island (BCI, Panama) and Yasuní (Ecuador) where density was lower and sampled individuals had ≥ 1.0 cm dbh (Table 2). Sampling ranges spanned ca. 1–4 km, in transect-like design following topographic features for ease of orientation, except in the forest monitoring sites of Paracou (French Guiana), BCI and Yasuní, where random sampling was conducted in established plots.

**Table 2:** Physical and ecological characteristics of sampled *Symphonia globulifera* populations. H_M-m_, maximum and minimum sampling altitude (m); T, annual mean temperature (°C); P, annual precipitation (mm); and D (d), density of *S*. *globulifera* stems ≥10 cm dbh (≥1.0 cm dbh) with d only available for BCI and Yasuni (stems/ha); for populations not corresponding to monitoring sites, the values are approximate estimates (~).

Population	Latitude	Longitude	H_M-m_	T	P	D (d)
**Neotropics**						
Barro Colorado Island, Panama	9.15	-79.85	149–196	25.9	2632	0.48 (3.12)
Yasuní, Ecuador	-0.68	-76.39	231–273	23.8	2380	0.68 (1.76)
Paracou, French Guiana	5.27	-52.93	38–67	22.0	2496	10.5
Ituberá, Brazil	-13.80	-39.18	92–164	25.8	2817	~ 6.55
**Africa**						
São Tomé, São Tomé and Principe	0.27	6.56	671–1896	23.8	2058	~ 1.43
Nkong Mekak, Cameroon	2.77	10.53	473–838	25.8	2837	~ 9.58
Mbikiliki, Cameroon	3.19	10.53	467–911	25.6	2806	~ 9.10

**Fig 2:**
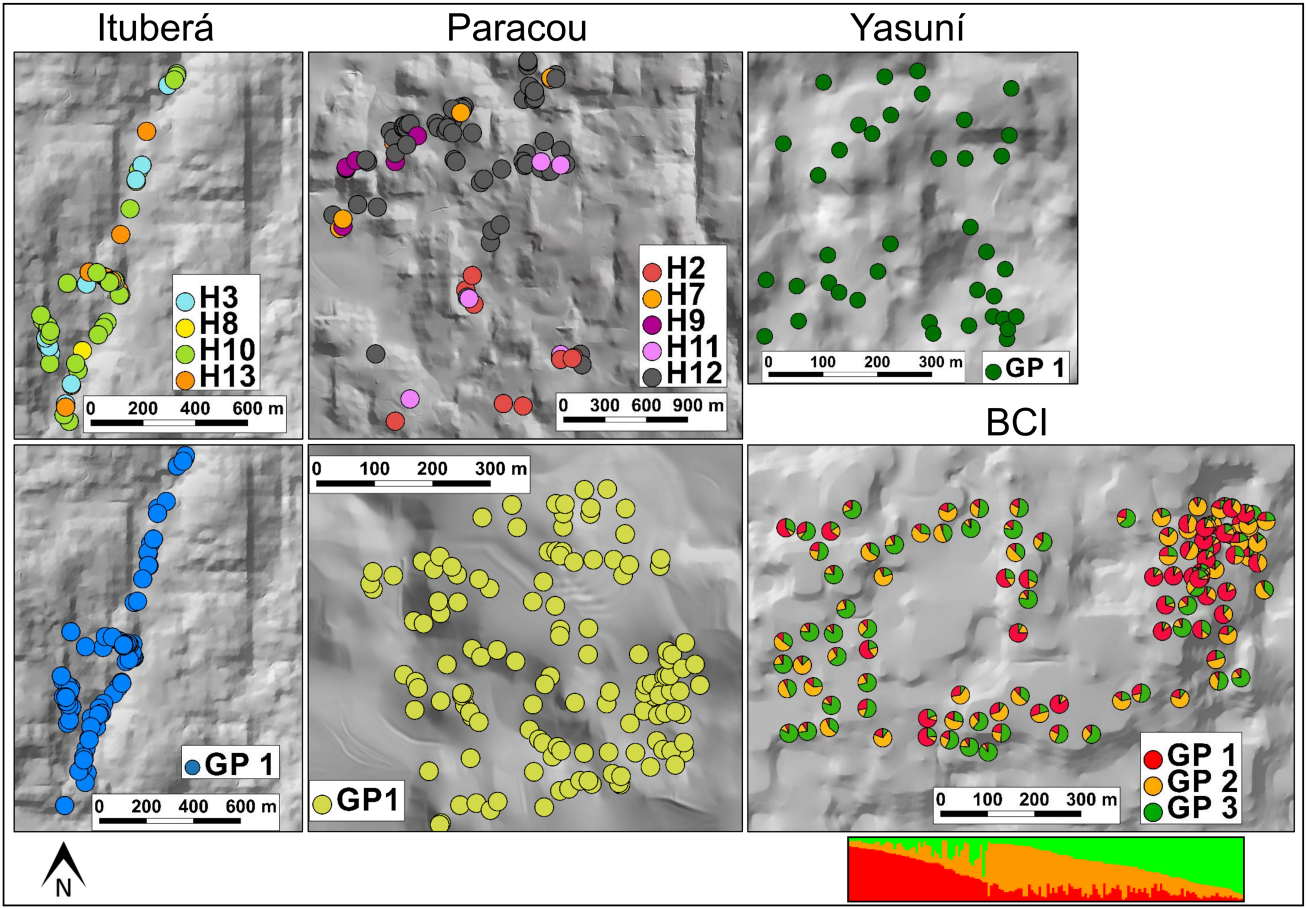
Fine-scale spatial genetic structure in Neotropical populations of *Symphonia globulifera*. Each individual is plotted on the map as a disc representing the colour of its specific plastid DNA haplotype (“H”) or as a pie chart indicating the ancestry proportions, *Q*, in different genetic clusters (“GP”), as defined in the STRUCTURE analysis for the number of clusters *K* best describing the data. Individual STRUCTURE barplots below each population map illustrate the distribution of ancestry proportion for each of the K gene pools.

**Fig 3:**
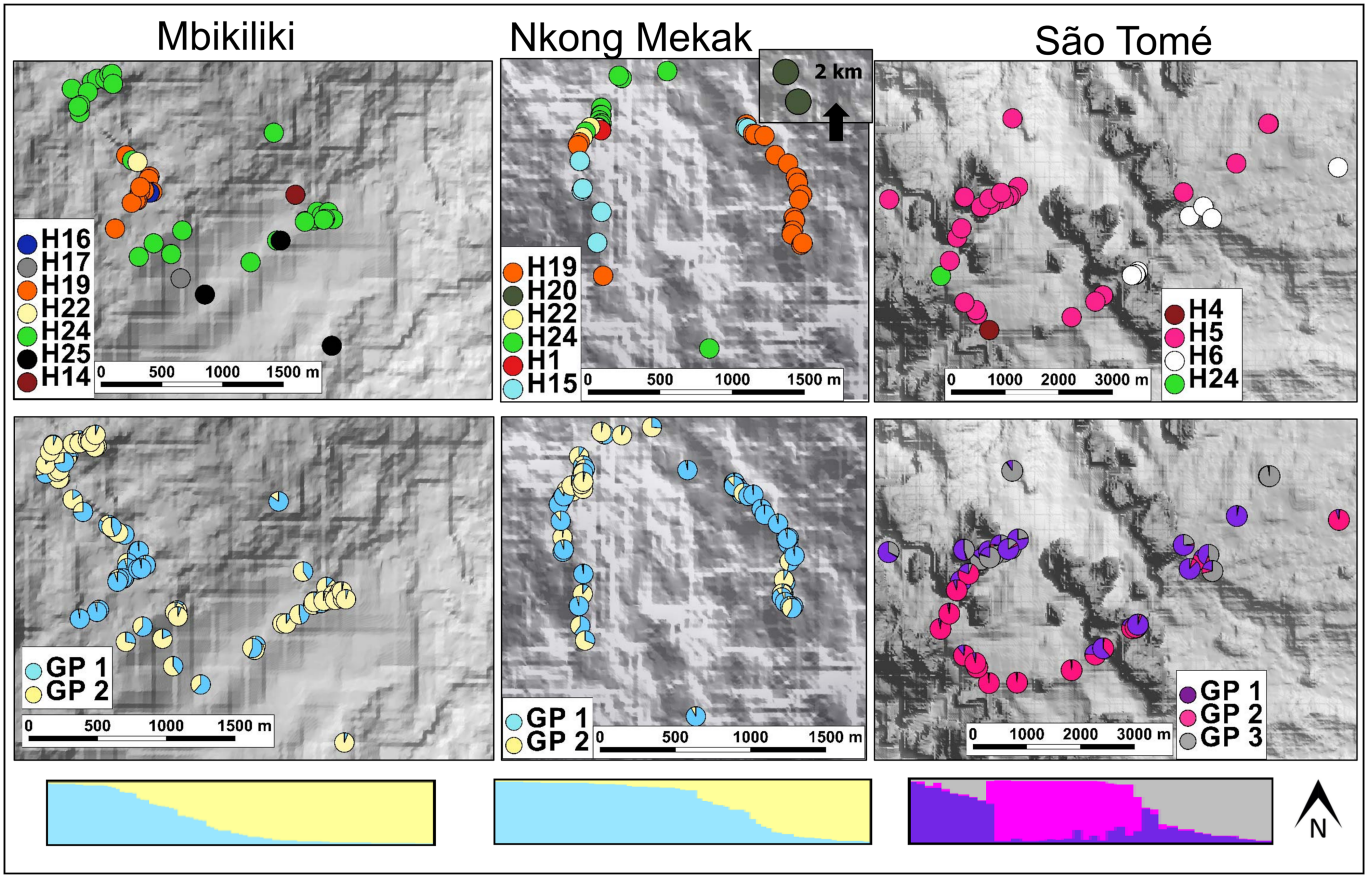
Fine-scale spatial genetic structure in African populations of *Symphonia globulifera*. Each individual is plotted on the map as a disc representing the colour of its specific plastid DNA haplotype (“H”) or as a pie chart indicating the ancestry proportions, *Q*, in different genetic clusters (“GP”), as defined in the STRUCTURE analysis for the number of clusters *K* best describing the data. Individual STRUCTURE barplots below each population map illustrate the distribution of ancestry proportion for each of the K gene pools.

The selected populations spanned a wide range of climatic (WorldClim 1.4 dataset [78]) and topographic (ASTER Global Digital Elevation Model, http://reverb.echo.nasa.gov/) conditions (Figs 2 and 3, Table 2). The altitudinal range of sampled populations was larger in Africa (365–1225 m) than in the Neotropics (25-72m). There was also a marked variation in dispersal vectors between continents (Table 1). In Paracou, samples included swamp and *terra firme* morphotypes. For this population, samples were collected for plastid DNA analysis, whereas SSR data (for different trees) was reanalysed from Degen et al. 2004 [72].

### Ethics statement

Our research complied with national and international legislation: research and sampling permits were obtained from the Ministry of Scientific Research and Innovation of Cameroon (59/MINRESI/B00/C00/C10/C13), from the responsible of the Paracou station (i.e. the French Agricultural Research Institute for Development, CIRAD), and from the managers of the BCI and Yasuní plots (i.e. the Smithsonian Tropical Research Institute). For Ituberá (Brazil), we obtained a sampling permit from the Chico Mendes Institute for Biodiversity (SISBIO 19053–1) and an export permit from the Ministério do Meio Ambiente, Brazil (Requerimento N° 107231).

### Molecular markers

DNA was extracted using the Qiagen DNeasy plant kit (Qiagen Corporation, Valencia, CA) or the Invisorb DNA Plant HTS 96 Kit (Invitek, Berlin, Germany). SSR data (five loci) were generated at the University of Michigan, Ann Arbor, USA, for populations BCI and Yasuní, as described in Dick and Heuertz [38], and at INIA-CIFOR, Madrid, Spain, for populations Ituberá, São Tomé, Nkong Mekak and Mbikiliki, following the protocols of Budde *et al*. [30]. SSR data for Paracou was taken from Degen *et al*. [72] and contained three loci. All SSR data were resolved on capillary sequencers (Applied Biosystems, Carlsbad, USA; see S1 File for experiment details and genotype matrices). The SSR loci of all populations belonged to a total set of six loci (Sg03 and Sg18 [72]; SgC4 and Sg19 [59]; Sg06 and Sg10 [79]), but were not exactly the same in each population because of variable amplification and genotyping success due to large genetic distances among some of the populations [38,80].

Sequences of the *psbA-trnH* plastid DNA (cpDNA) intergenic spacer were generated for random subsamples from Paracou, Ituberá, São Tomé, Nkong Mekak and Mbikiliki, completing the data sets of Dick and Heuertz [38] and Budde *et al*. [30] (see sample sizes in Table 2). Amplification with *psbAF* and *trnHR* primers [81] was performed at INIA-CIFOR as in Budde *et al*. [30] with a modified PCR profile: 30 s at 98°C, 35 cycles of 5 s at 98°C, 10 s at 50°C and 35 s at 72°, and a final elongation of 3 min at 72°C. PCR products were purified using Exonuclease I and Calf Intestinal Alkaline Phosphatase (New England Biolabs) and sequenced using the services of Macrogen Europe (The Netherlands). Sequences were assembled, edited and aligned in CodonCode Aligner 4.2.5 (CodonCode Corporation, Dedham, MA, USA).

### Data analysis

#### Genetic diversity

The rarefied allelic richness (*A*_R_) and the expected heterozygosity corrected for sample size (*H*_E_) were computed for nuclear SSRs using SPAGeDi 1.4c [82]; the standard error of *H*_E_ was estimated from jackknife replicates using the PopGenKit package [83] in *R* version 3.1.1 [84]. To assess deviations from Hardy-Weinberg genotypic proportions, e.g. caused by non-random mating or null alleles, we computed the fixation index (*F*_IS_) and tested deviation from zero using 10,000 permutations of alleles within populations in SPAGeDi. We estimated the frequencies of possible null alleles using the Brookfield2 estimator [85,86] and estimated a fixation index corrected for null alleles in MicroChecker 2.2.3 [87]. Occasionally, multilocus genotypes were found more than once within populations. The probability for these copies to be derived from distinct sexual reproductive events, *psex*, was computed in GenClone [88]. Plastid haplotypes were defined combining nucleotide polymorphisms, indels and inversions in the sequence. Rarefied plastid haplotye richness, *A*_Rp_, and haplotypic diversity, *h*, were obtained in SPAGeDi 1.4c.

#### Test and quantification of FSGS

To test for the presence of overall FSGS in each population and quantify its strength, we followed the approach of Vekemans and Hardy [1] for nuclear and plastid DNA markers separately. Pairwise kinship coefficients *F*_ij_ [89] were calculated in SPAGeDi 1.4c in all populations and were regressed on the logarithm of pairwise spatial distances between individuals. The significance of the regression slope *b* was tested using 10,000 permutations of the spatial position of the individuals. The strength of FSGS was estimated as *Sp = -b/(1-F*_ij(1)_*)* [1] where *F*_ij(1)_ is the average kinship coefficient of individuals in the first distance class. The number and size of distance classes was defined for each population according to recommendations from the SPAGeDi user manual [90]: similar numbers of pairwise comparisons across classes, > 50% of individuals present in each class and a coefficient of variation < 1 of the number of times each individual was represented in each class.

As an alternative to *Sp*, we also performed a spatial principal component analysis (sPCA) in the *adegenet* package in *R* [91]. This allowed us to test for overall FSGS (e.g. patches of related individuals, allele frequency gradients) using a G-test, and to estimate the strength of spatial structure as the eigenvalue of the first sPCA axis, *eig*.*sPCA*. Since we observed *a priori* a stronger FSGS in African than Neotropical populations, we assessed differences in FSGS between continents using T-tests based on either *Sp* values or *eig*.*sPCA*. To specifically address the relationship between FSGS and altitudinal sampling range within populations, we performed a Spearman rank correlation test between *Sp* or *eig*.*sPCA* and the standard deviation of sampling altitude using *R* (see also next section).

#### Spatial genetic heterogeneity and its causes

Besides FSGS due to drift-dispersal equilibrium, non-equilibrium processes such as selection or barriers to reproduction can lead to spatial genetic heterogeneity (SGH). We tested for SGH using two types of approaches based on nuclear SSRs, and then investigated its potential causes through examining its strength, congruence with SGH at maternally inherited plastid markers and specific spatial arrangement. For SSRs, we used: 1) a G-test to detect global structure (see above) and an L-test to detect local structure, the latter corresponding to an increased differentiation between spatially close individuals, and estimated sPCA scores [−1,1] for each individual on the first global or local sPCA axes, respectively [27]; 2) the Bayesian clustering analysis implemented in STRUCTURE ver. 2.3.4 [92] to detect sympatric gene pools (GPs) and estimate ancestry proportions (*Q*, [0,1]) for each individual in each GP. STRUCTURE was chosen because it is particularly efficient at detecting GPs that co-occur in the same geographical site when spatial structure is weak [24] and because it allows to test whether a model with differentiated GPs (*K*>1) fits the data better than a model with a single GP (*K* = 1). To detect shared GPs among populations, STRUCTURE was run separately for the groups of populations that were genotyped together (to avoid mixing SSRs datasets for which allele identities were not cross-standardised: Ituberá and African populations; Yasuní and BCI). Then the analysis was run within each population. We used an admixture model with correlated allele frequencies for codominant markers and 10 repetitions for each number of clusters, *K*, from 1 to 7, using a burn-in length of 20,000 and a run length of 80,000 iterations. Chain convergence was checked visually. The *K* that best described the data was determined as the one with the highest logarithm probability of data, ln Pr(X|*K*) (also referred to as L(*K*)), following Pritchard *et al*. [93], and using the Delta *K* (Δ*K*) method described by Evanno *et al*. [94] (see S2 File). Since the codominant markers model is not necessarily robust to the effect of null alleles, we repeated all analyses using the recessive alleles model as explained in the STRUCTURE documentation to assess the effect of null alleles on the clustering solution. Also, for sites with *K >* 1, analyses were repeated using an admixture model in the spatially explicit clustering program TESS 2.3.1. [24] using either a constant or a linear trend, and for the latter, a spatial interaction parameter of 0.6 or 1 (S2 File). Maps of the geographical distribution of GPs and haplotypes per sampling location were built in QGIS 2.4 [95].

If within-population SGH occurs, its strength, its association with plastid DNA and its spatial arrangement, e.g. in the context of habitat variation, can provide further information on its ecological and evolutionary determinants. As measures of SGH, we used the individual sPCA scores and GP ancestry proportions (*Q*) computed above. First, to examine the strength of SGH, we assessed *Q* values and genetic differentiation (*F*_ST_) among co-occurring GPs, considering higher *Q* and higher *F*_ST_ as indicators of stronger divergence. *F*_ST_ among GPs within populations was estimated and tested with permutation tests using SPAGeDi, assigning individuals to GPs based on *Q*≥50% or *Q*≥87.5%, the latter category susceptible to include genetically pure, first and later-generation backcrosses [96]. Within GPs based on *Q*≥87.5%, e.g. representing putative distinct reproductive demes, we assessed the presence of null alleles, deviation from Hardy-Weinberg proportions and strength of FSGS as above. Second, to test for historically diverged lineages, we assessed cyto-nuclear disequilibria [97] between plastid haplotypes and nuclear SGH. We performed one-way ANOVA in *R* to detect if groups of individuals carrying the same haplotype differed in their mean individual sPCA scores. We also tested for association of haplotypes and GPs (*Q*≥50%) using Fisher tests that ignored spatial autocorrelation in the data and we performed partial Mantel tests based on similarity matrices (“1” for pairs of individuals sharing the same haplotype or GP, “0” for pairs with different haplotypes or GPs) in which we controlled for spatial autocorrelation through a spatial distance matrix using the Ecodist package [98] in *R*. In Paracou, we further tested the association between GPs or haplotypes and morphotypes, similarly using Fisher and partial Mantel tests. Third, we examined altitudinal stratification of genetic variation in each population as would be expected, for example, in the case of restricted mobility of dispersers due to slopes [99,100]. We performed one-way ANOVA to test if three *ad hoc* defined altitudinal classes differed in their mean individual sPCA scores, and in their mean *Q* value for each GP. Association with altitudinal classes was preferred over a regression analysis because the relationship to test is not necessarily linear.

## Results

### Genetic diversity

Nuclear microsatellite data and individual coordinates are reported in S1 File. The number of SSR alleles per locus and population ranged from three to 35. In Ituberá, four multilocus genotypes (genets) occurred in more than one (2–3) trees (ramets) sampled in close proximity (4-25m), and in São Tomé, two trees carried the same genotype, but spatial coordinates were unknown for one copy. Haplotype data was only available for both genotype copies in São Tomé, which bore identical haplotypes. *Psex* for the genotype copies was low, from 1.13 × 10^−8^ to 2.17 × 10^−4^, suggesting that trees with identical multilocus genotypes represented clonal copies. Heterozygosity and allelic richness estimates were high and similar in all populations with the exception of Ituberá, where both statistics were slightly lower, although not significantly different from other populations (Table 3). Significant inbreeding was detected in all populations but Yasuní (Table 3). Microchecker detected null alleles in all inbred populations, but *F*_IS_ corrected for null alleles remained significant in several populations, especially in Africa, suggesting non-random mating (Table 3).

**Table 3:** Genetic diversity estimates of *Symphonia globulifera* populations. n_nuc_, sample size for SSR data; SSR, number of SSR loci genotyped; *A*, mean number of alleles per locus; *A*_R_ (SD), allelic richness or number of alleles expected in a sample of 34 individuals and its standard deviation; *H*_E_ (SE), expected heterozygosity and its standard error based on jackknife resampling; *F*_IS_, fixation index; *F*_IS_*, fixation index after null allele correction; n_cp_, sample size for plastid DNA; hap, number of plastid haplotypes; *A*_Rp_, plastid haplotype richness or number of haplotypes expected in a sample of 10 individuals; *h*, gene diversity for plastid haplotypes corrected for sample size. ***, *P*≤0.001; **, *P*≤0.01; *, *P*≤0.01; ns, not significant; nc, not computed.

Population	n_nuc_	SSR	*A*	*A*_R_ (SD)	*H*_E_ (SE)	*F*_IS_	*F*_IS_ *	n_cp_	hap	*A*_Rp_	*h*
**Neotropics**											
BCI	147	5	13.8	8.68 (2.07)	0.831 (0.016)	0.148***	-0.049**	10	2	2	0.356
Yasuní	34	5	11	9.21 (2.46)	0.783 (0.038)	0.057ns	nc	10	1	1	0
Paracou	148	3	23.6	12.57 (3.56)	0.880 (0.031)	0.172***	-0.003^ns^	96	5	3.21	0.494
Ituberá	85	5	10.8	7.26 (5.10)	0.632 (0.061)	0.107***	0.072*	50	4	3.03	0.594
**Africa**											
São Tomé	42	5	12.6	9.81 (2.76)	0.813 (0.022)	0.183***	0.111***	38	4	2.48	0.450
Nkong Mekak	70	5	14.2	9.95 (5.59)	0.801 (0.044)	0.148***	0.081***	49	6	4.04	0.729
Mbikiliki,	94	5	16.2	9.81 (4.82)	0.748 (0.043)	0.154***	0.086***	50	7	3.34	0.571

Twenty-five plastid DNA haplotypes were detected across populations. The *psbA-trnH* alignment varied at 28 positions, for sequence lengths of 289–515 bp. Polymorphism varied strongly between populations, from one haplotype in Yasuní to 6 and 7 haplotypes in the Cameroonian populations (Table 3, see S3 File for the complete list of Genbank accession numbers, including the newly generated sequences KX572421—KX572686), a result that was mirrored in the estimates of rarefied haplotype richness (Table 3).

### Fine scale spatial genetic structure (FSGS)

Significant FSGS was observed in all populations except Yasuní, with an estimated strength of FSGS from *Sp* = 0.0003 in Yasuní to *Sp* = 0.0341 in São Tomé (Table 4, Fig 1). *Sp* values and their significance remained similar when the analysis was restricted to three loci in all populations (the loci assessed by Degen *et al*. [72] in Paracou, S4 File). These results suggested that the analysed SSRs had sufficient power to detect FSGS and estimate its strength. The sPCA analysis identified a significant global FSGS by means of a G-test within all populations, with eigenvalues of the first sPCA axis, *eig*.*sPCA*, ranging from 0.029 in BCI to 0.272 in São Tomé (Table 4). FSGS was stronger in African than in Neotropical populations with mean *Sp* = 0.025 in Africa *vs*. 0.008 in America and mean *eig*.*sPCA* = 0.180 in Africa *vs*. 0.049 in America (*P* = 0.029 for *Sp* and *P* = 0.014 for *eig*.*sPCA* using one-tailed T-tests). *Sp* and *eig*.*sPCA* were both positively correlated with altitudinal sampling range (for *Sp*: Spearman rho = 0.76, *P* = 0.033; for *eig*.*sPCA*: rho = 0.89, *P* = 0.006).

**Table 4:** Estimates of FSGS parameters in *Symphonia globulifera* populations. n_nuc_, sample size for SSR data; n_cp_, sample size for plastid DNA; DC, number of distance classes; 1st DC, maximum distance of the first class (m); *F*_ij(1)_, mean kinship coefficient of the first distance class; *Sp*, intensity of FSGS and *P*-value of one-sided test of the regression slope *b* of *F*_*ij*_ on the logarithm of spatial distance; *b* (SE), jackknife mean of *b* and its standard error; *eig*.*sPCA*: eigenvalue of the first sPCA axis and significance of G-test. ns, not significant. ***, *P*≤0.001; **, *P*≤0.01; nc, not calculated (no coordinates available).

	*SSRs*							*Plastid DNA*		
Population	n_nuc_	DC	1^st^ DC	*F*_*ij(1)*_	*Sp*	*b* (SE)	*eig*.*sPCA*	n_cp_	*Sp*	*b*
**Neotropics**										
BCI	147	7	113	0.030	0.0166***	-0.0161 (0.0060)	0.029***	10	nc	nc
Yasuní	34	6	131	0.003	0.0003ns	-0.0003 (0.0057)	0.057***	10	nc	nc
Paracou	148	4	203	0.015	0.0090***	-0.0088 (0.0029)	0.038***	96	0.1021***	-0.0925
Ituberá	85	5	152	0.009	0.0074**	-0.0074 (0.0023)	0.073***	50	-0.0032^ns^	0.0032
**Africa**										
São Tomé	42	6	856	0.084	0.0341***	-0.0312 (0.0096)	0.272***	38	0.4951***	-0.2802
Nkong Mekak	70	5	312	0.020	0.0124***	-0.0122 (0.0034)	0.111***	49	0.2769***	-0.1787
Mbikiliki	94	7	240	0.072	0.0273***	-0.0253 (0.0105)	0.154***	50	0.4883***	-0.2069

Most populations displayed significant FSGS for maternally inherited plastid DNA sequences with *Sp* ranging from -0.0032 to 0.4883. The signal was an order of magnitude greater than at nuclear markers, with stronger structure in African (*Sp* ≥ 0.277) than in Neotropical populations (*Sp* ≤ 0.102, Table 4).

### Spatial genetic heterogeneity and its causes

Based on sPCA, we detected global structure in all populations (G-test, see above), but the L-test for local structure was not significant in any population, suggesting that neighbouring individuals were not strongly differentiated. STRUCTURE analysis for codominant markers across populations revealed that each population segregated into its own GP except the two Cameroonian populations, which shared the same two GPs. Within populations, the number of GPs that best explained the data was *K* = 1 in the American populations Yasuní and Ituberá, and *K* = 2 or *K* = 3 in the African populations (Figs 2 and 3, Table 5, S2 File). For the American populations Paracou and BCI, the selection of the best *K* was not trivial: STRUCTURE barplots reflected subtle substructure with uneven ancestry proportions *Q* across individuals in up to *K* = 3 clusters, but L(*K*) was highest for *K* = 3 in BCI and for *K* = 1 in Paracou, the solutions we eventually retained (S2 File). The recessive alleles model in STRUCTURE gave similar results, with Pearson correlation coefficients *r*≥0.94 for individual ancestry proportions between the codominant and recessive alleles models (S2 File). We thus considered that null alleles had a negligible effect on the STRUCTURE analysis, and retained only results from the codominant marker model for further analyses. In populations with multiple GPs, the proportion of individuals assigned at *Q*>0.875 was high (57–70%) in the African populations reflecting putative coexisting demes, while individuals in American populations were more homogeneous or admixed on average (Table 5; compare bar plots representing *Q* in American [Fig 2] vs. African populations [Fig 3], S2 File). STRUCTURE results were broadly congruent with those obtained in TESS (S2 File).

**Table 5:** Strength of genetic differentiation between nuclear gene pools (GPs) within *Symphonia globulifera* populations. *K*, number of STRUCTURE clusters; *F*_ST(*Q*≥0.5)_, *F*_ST_ among GPs with individual assignment based on *Q*≥ 0.5; *F*_ST(*Q*≥0.875)_, *F*_ST_ among GPs with *Q*≥0.875; PI50 (%), proportion of individuals assigned to a GP based on *Q*≥ 0.5; PI87 (%), proportion of individuals assigned to a GP based on *Q*≥0.875. nd, not defined; ***, *P*≤0.001.

Population	*K*	*F*_ST(*Q*≥0.5)_	*F*_ST(*Q*≥0.875)_	PI50 (%)	PI87 (%)
**Neotropics**					
BCI	3	0.082***	nd	76.9	0
Yasuní	1	nd	nd	nd	nd
Paracou	1	nd	nd	nd	nd
Ituberá	1	nd	nd	nd	nd
**Africa**					
São Tomé	3	0.168***	0.234***	97.6	57.1
Nkong Mekak	2	0.082***	0.141***	100.0	70.0
Mbikiliki	2	0.102***	0.188***	100.0	67.0

In the African populations, *F*_IS_ within GPs (*Q*≥0.875) was generally non-significant (S1 Table), suggesting that deviation from Hardy-Weinberg equilibrium at the population level (Table 3) was largely due to population substructure. *F*_IS_ was however significant in GP2 in both Mbikiliki and Nkong Mekak and remained significant after correction for null alleles in Nkong Mekak, suggesting deviation from random mating within this GP, e.g. due to selfing or biparental inbreeding.

Cyto-nuclear disequilibria based on ANOVA were detected in the three African populations only: individuals carrying different haplotypes differed in their mean sPCA score (Table 6). Haplotype-GP association tests were only significant in the two Cameroonian populations, where GP1 was associated with haplotype H19 and GP2 with H24 in both populations (Fisher test, *P*<0.001; Fig 2). The associations were still significant after controlling for geographical distance (partial Mantel tests, *P*<0.001 in both populations). In Paracou, individuals from the same morphotype were genetically more related at plastid DNA than expected at random (Fisher test: *P*<0.01; partial Mantel test: *P*<0.05).

**Table 6:** Spatial genetic heterogeneity in SSR data and its association with plastid DNA haplotypes, i.e. cytonuclear disequilibria, and altitude. The mean sPCA score for the first sPCA axis is given for individuals carrying the same haplotype, sPCA (hap), or belonging to the same *ad hoc* altitudinal class sPCA (alt); n, sample size range per altitudinal class. *P* values represent the significance of ANOVA analyses testing differences in the mean sPCA score for haplotypes, *P*(hap) or altitudinal classes, *P*(alt). nc^1^, not computed because coordinates were unavailable or populations were monomorphic; nc^2^, not computed because SSR and plastid DNA data were collected from different individuals; ns, not significant; ***, *P*≤0.001; **, *P*≤0.01; *, *P*≤0.05.

Population	*P*(hap)	sPCA (hap1)	sPCA (hap2)	sPCA (hap3)	*P*(alt)	n	sPCA (alt1)	sPCA (alt2)	sPCA (alt3)
**Neotropics**									
BCI	nc^1^	nc^1^	nc^1^	nc^1^	ns	46–51	-0.022	-0.011	0.031
Yasuní	nc^1^	nc^1^	nc^1^	nc^1^	ns	11–12	0.053	0.003	-0.057
Paracou	nc^2^	nc^2^	nc^2^	nc^2^	ns	46–55	0.036	-0.044	0.001
Ituberá	ns	-0.086 (H10)	-0.104 (H13)	0.091 (H3)	ns	28–29	0.127	-0.071	-0.055
**Africa**									
São Tomé	*	-0.209 (H5)	0.431 (H6)	-	***	12–16	-0.568	0.036	0.716
Nkong Mekak	***	-0.412 (H15)	-0.309 (H19)	0.323 (H24)	**	23–24	-0.075	0.300	-0.216
Mbikiliki	***	-0.688 (H19)	0.182 (H25)	0.258 (H24)	***	31–32	0.230	-0.335	0.102

Finally, we detected a clear altitudinal stratification in African but not in Neotropical populations: in Africa, individuals from different *ad hoc* altitude classes differed in their mean sPCA scores (Table 6). Further, in all populations with multiple GPs, at least one GP was associated with a specific altitudinal class (S2 Table). These results also supported stronger altitudinal stratification of GPs in Africa.

## Discussion

Six out of seven *Symphonia globulifera* populations from Africa and America displayed fine-scale spatial genetic structure based on *Sp*, and all seven had a significant FSGS based on sPCA. The magnitude of FSGS was overall in agreement with expectations for outcrossed tropical trees but varied strongly among populations, from *Sp* = 0.000 to *Sp* = 0.034 for SSRs. African populations had a much stronger FSGS signal than Neotropical populations, based on both nuclear and plastid markers, and the signal was associated with larger altitudinal gradients in Africa than in America. These results suggested on average a more restricted gene flow, and especially a more restricted seed-based gene flow, in African than in American populations, reflecting a more restricted movement of dispersers in rugged African populations. There was limited evidence for selfing in *S*. *globulifera*, but null alleles and population substructure (SGH) contributed to deviations from Hardy-Weinberg genotypic proportions within populations. There was evidence for cyto-nuclear disequilibria and historical gene pool differentiation in the two Cameroonian populations, while the population from French Guiana displayed an association of plastid haplotypes with two morphotypes characterized by differential habitat preferences.

### Methodological considerations

Some methodological issues are worth discussing with regard to our results. First, samples were collected either randomly or following approximate transects in different populations (Figs 2 and 3). This should not have affected meaningfully the estimation of FSGS using the *Sp* statistic, which is robust towards differences in sampling scheme [1,101]. Second, our sampling covered large distances (maximum distance of one to several km) within populations, likely covering the suitable distance range where kinship decays linearly with the logarithm of distance [1], hence minimizing the risk of overestimating FSGS due to too short sampling distances [102]. Third, our sampling scheme had probably a low power to estimate the decay of kinship at short distance because only a low proportion of sample pairs corresponded to true nearest neighbours in the populations. Forth, our populations featured different densities of *S*. *globulifera*. This can affect FSGS, which is expected to increase in low-density populations because of a reduced overlap of seed shadows [1,7]. Against *a priori* expectations, however, weak FSGS was observed in the low-density Neotropical populations BCI and Yasuni, where sampling included also younger individuals (>1cm dbh) potentially representing cohorts of related individuals. The weaker than expected FSGS in these populations could have been caused by confounding factors, e.g. increased animal-mediated dispersal distance in low-density populations [12]. In any case, considering the stronger FSGS in African than in Neotropical populations observed in our study, variation in population density and age of sampled individuals did not appear to magnify the pattern of FSGS differences among populations.

Although the number of SSRs used in our study was low (3–5), these highly polymorphic markers were able to detect significant FSGS in all and in six out of seven populations, by means of the G-test and *Sp*, respectively. This indicates a sufficient power for the purpose of the study. In fact, a dataset of 18 genic SSRs on ca. 30 individuals [80] had a lower power than our FSGS analyses (FSGS analyses on data from Olsson *et al*. 2016 are reported in S4 File). Another risk of using a low marker number is that it can lead to erroneous GP inference (e.g. using STRUCTURE) because few markers do not capture well the diversity of stochastic lineage sorting processes due to random genetic drift [103]. To mitigate this potential problem, we used two types of cyto-nuclear and habitat association analyses, i.e. based on GPs and based on sPCA scores, which gave congruent results. Several studies also reported that IBD can lead to overestimation of the number of GPs inferred by STRUCTURE [22,104]. Explicitly adjusting for IBD in our populations by using a spatial prior in the TESS analysis did however not reduce the number of inferred GPs (S2 File). Our results suggest that ancestry proportions *Q* should complement the interpretation of *K* because *K* alone does not characterize population substructure well. Examining both statistics in the populations where *K* = 3, we can interpret GPs in São Tomé as putative distinct demes with some degree of reproductive isolation, whereas in BCI, we conclude that GPs are mostly a result of allele frequency gradients (Table 5).

### Biotic and abiotic determinants of within-population spatial genetic structure

The observed FSGS patterns in our study can be explained through a series of factors, including topographic complexity, seed and pollen dispersal features, biogeographic history and, potentially, microenvironmental adaptation.

At SSRs, FSGS was in the range expected for species with outcrossing or mixed mating systems (*Sp* from 0.0126 to 0.0372) and animal- or gravity-mediated seed dispersal (*Sp* from 0.0088 to 0.0281, [1,8,105]), in agreement with *S*. *globulifera*’s life history traits [59,73]. *Sp* at maternally inherited plastid DNA was generally an order of magnitude greater than at biparentally inherited SSRs. This pattern suggests that, among other factors, restricted seed dispersal shapes FSGS in *S*. *globulifera* whereas pollen is the long-distance component of gene flow (see [30]), a typical pattern in tropical trees (e.g. [8,29]).

The factor that most clearly co-varied with FSGS was altitudinal sampling range: stronger FSGS was observed in populations sampled in more prominent altitudinal gradients, specifically, in African populations with large altitudinal gradients (>350 m in Cameroon, >1200 m in São Tomé). Steep topography is known to restrict the mobility of animal species [99,100,106], thus reducing gene flow and increasing the genetic structure of the plants that these animals disperse [14,15]. This mechanism could partially explain the strong FSGS in African populations of *S*. *globulifera*. In addition, the complexity of habitats and the vegetation associated with such gradients could have favoured microenvironmental adaptation of *S*. *globulifera* (see below) and/or specialization of its dispersers, in terms of behaviour or community composition [107,108], restricting seed dispersal. Unfortunately, no data on the precise composition of disperser communities or the behaviour of *S*. *globulifera* dispersers are available for our study populations. On the other hand, large-scale differences in disperser communities between continents can contribute to explaining the observed pattern. Bats (*Artibeus spp*.) or tapirs (*Tapirus terrestris* and *Tapirus bairdii*), endemic to the Neotropics, can disperse propagules from hundreds of meters to several kilometres ([61,62,66,109], see Table 1). Both bats and tapirs (the latter not present in BCI) can concentrate a wide spectrum of seed genotypes at their feeding roosts or latrines, respectively, promoting seed shadow overlap and thereby, decreasing FSGS [59,71]. Bat-mediated seed dispersal of *S*. *globulifera* has only been reported in American populations, although frugivorous bats occur also in Africa. Hornbills could constitute an equivalent long-seed disperser in Africa but they putatively regurgitate the seeds of *S*. *globulifera* [49], which could reduce dispersal distances compared to endozoochory [50,110,111].

The two Cameroonian populations Mbikiliki and Nkong Mekak provided an interesting example of biogeographic history shaping within-population structure. In these populations, we observed the same associations between GPs and plastid haplotypes, and evidence of preferential reproduction within GPs (Wahlund effect). Such cyto-nuclear associations reflect the sympatric occurrence of differentiated lineages. An allopatric differentiation of such lineages is most commonly proposed, for instance in distinct refugia where rainforest species persisted during the dry and cold periods of the Pleistocene [112]. Cameroonian *S*. *globulifera* lineages now co-occur in the Ngovayang massif, a region that corresponds to a proposed Pleistocene refuge area [112–114]. A comparison with plastid haplotypes widely sampled across Lower Guinea (Gabon and Cameroon) suggested a restricted distribution of the concerned lineages, in agreement with previous suggestions of local population persistence and absence of evidence for pronounced range shifts in *S*. *globulifera* [30]. The cyto-nuclear disequilibria are thus unlikely to reflect insufficient time for genetic homogenization after colonization (e.g. [98,115]). Rather, we believe they reveal a persistent historical or adaptive pattern maintained by partial reproductive isolation or assortative mating [97,116].

Adaptation to locally heterogeneous habitats, e.g. to specific soil properties or associated vegetation, could also explain the genetic clustering and altitudinal stratification of GPs in our study ([19,117,118], see [119] for an overview). In Paracou, where soil moisture content decreases with relative elevation, Allié and collaborators [120] showed that the common *S*. *globulifera* morphotype is associated with moist valley bottoms whereas the alternative morphotype preferentially grows in the upper part of slopes. The morphotype—haplotype association in our data and the morphotype—GP association based on genic SSRs (S4 File) indicates that differential habitat preferences are paralleled by genetic differentiation in *S*. *globulifera* in Paracou. Similarly, local-scale genetic differentiation in the Neotropical tree *Eperua falcata* has been attributed to edaphic specialization [118,121]. Signals of microenvironmental selection can be detected in neutral markers [20,117,122] when they are linked to makers under selection, or when emerging reproductive barriers foster linkage among physically unlinked markers [21,123]. Adaptive divergence can thus potentially lead to cyto-nuclear disequilibria resulting in patterns like those observed in the Cameroonian populations.

Assortative mating can interact with other forces to enhance genetic structure, potentially resulting also in significant inbreeding. Mass flowering events and asynchronous flowering promote pollinator movements between flowers of the same tree (see [75,124,125]) leading to temporal assortative mating [126]. This is likely in *S*. *globulifera* which may produce up to 200 open flowers per tree each day and for which unsynchronized flowering is suspected [52,72,74]. Further, agamospermy (seed development without fertilization) has been observed in other Clusiaceae (e.g. in the genera *Garcinia* and *Clusia* [127–129]) and leads to groups of genetically identical individuals as observed in two of the studied *S*. *globulifera* populations. However, additional data is needed to determine whether the observed clonality is due to agamospermy or to root suckers.

## Conclusions

We detected a wide diversity of FSGS patterns within *S*. *globulifera* populations, from non-significant or weak FSGS in Neotropical populations to pronounced structure in African ones. The strength of FSGS correlated with both disperser communities and altitudinal sampling range, while our data also contained evidence for co-occurrence of differentiated lineages and GP aggregation following habitat features. These results highlight the importance of spatially explicit eco-evolutionary processes in the local habitat exploitation of an ancient tropical tree species. The microenvironmental scale thus seems crucial for evolutionary processes in persistent populations of tree species, as has recently been shown in reports on microenvironmental adaptation in forest trees [118–120,122].

## Supporting information

S1 File Geographic coordinates and microsatellite genotypes of *Symphonia globulifera* samples used in this study. (XLSX)

S2 File Genetic clustering based on STRUCTURE and TESS. (DOCX)

S3 File Evolutionary relationships among *psba-trnH* plastid DNA haplotypes and Genbank accession numbers of sequences. (DOCX)

S4 File Genetic diversity and spatial genetic structure statistics in *Symphonia globulifera* based on different groups of SSRs. (DOCX)

S1 Table Estimates of mating system and FSGS parameters in genetic clusters of *Symphonia globulifera*. (DOCX)

S2 Table Altitudinal clustering of gene pools in *Symphonia globulifera* populations. (DOCX)
