# Supplementary material for: Altitudinal gradients, biogeographic history and microhabitat adaptation affect fine-scale spatial genetic structure in African and Neotropical populations of an ancient tropical tree species
Source: PLoS One. 2017 Aug 3;12(8):e0182515. doi: 10.1371/journal.pone.0182515 (PMC5542443; doi:10.1371/journal.pone.0182515)
Supplement: S2 File — (DOCX) [file pone.0182515.s002.docx]

**S2 File. Genetic clustering based on STRUCTURE and TESS**

**Codominant marker model in STRUCTURE**

Figure S2.1. Illustration of the best number of genetic clusters *K* in STRUCTURE analyses (codominant marker model) for the African and American populations; results for *K* = 1 to *K* = 4. The best *K* was supported based on model log likelihood (L(*K*)) and Delta *K* (∆*K*). The consensus barplot for each K from 10 independent runs confirmed the selection visually*.*

**a) Mbikiliki & Nkong Mekak, *K*=2**

**b) São Tomé, *K*=3**

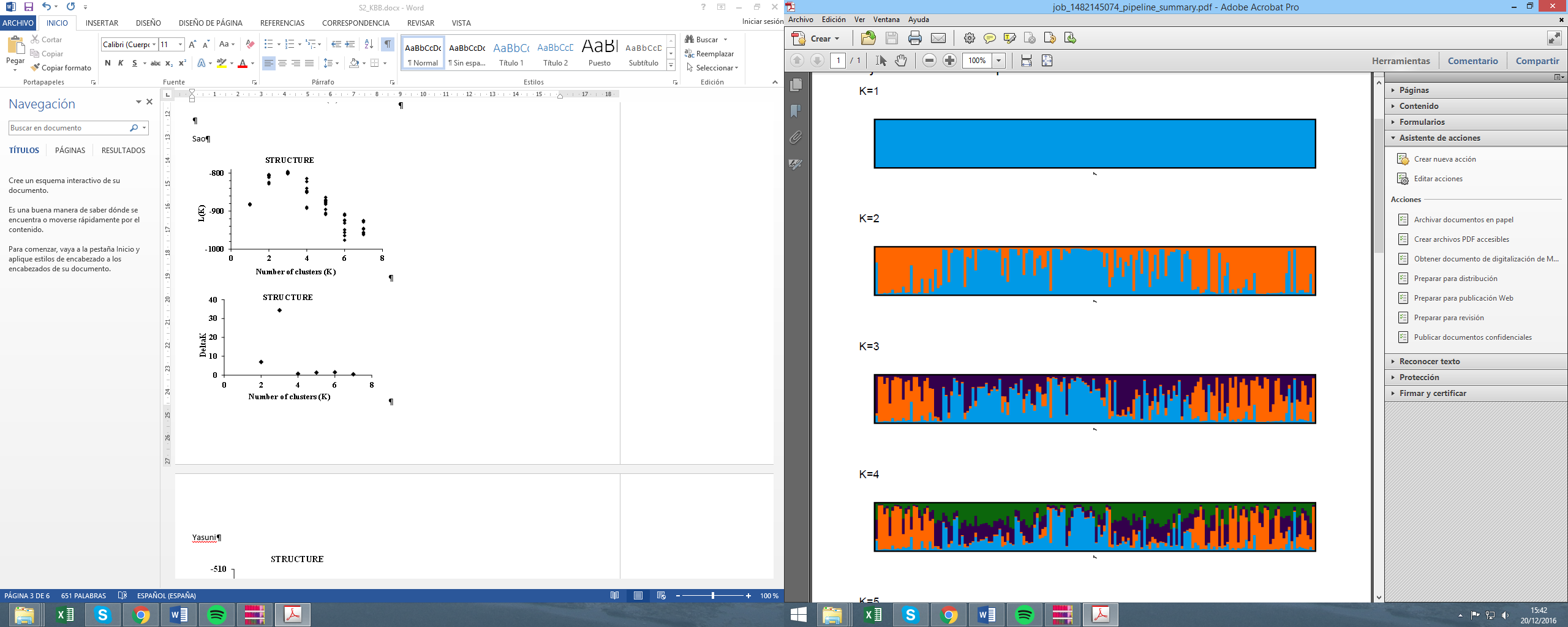


**K=1**

**K=2**

**K=3**

**K=4**


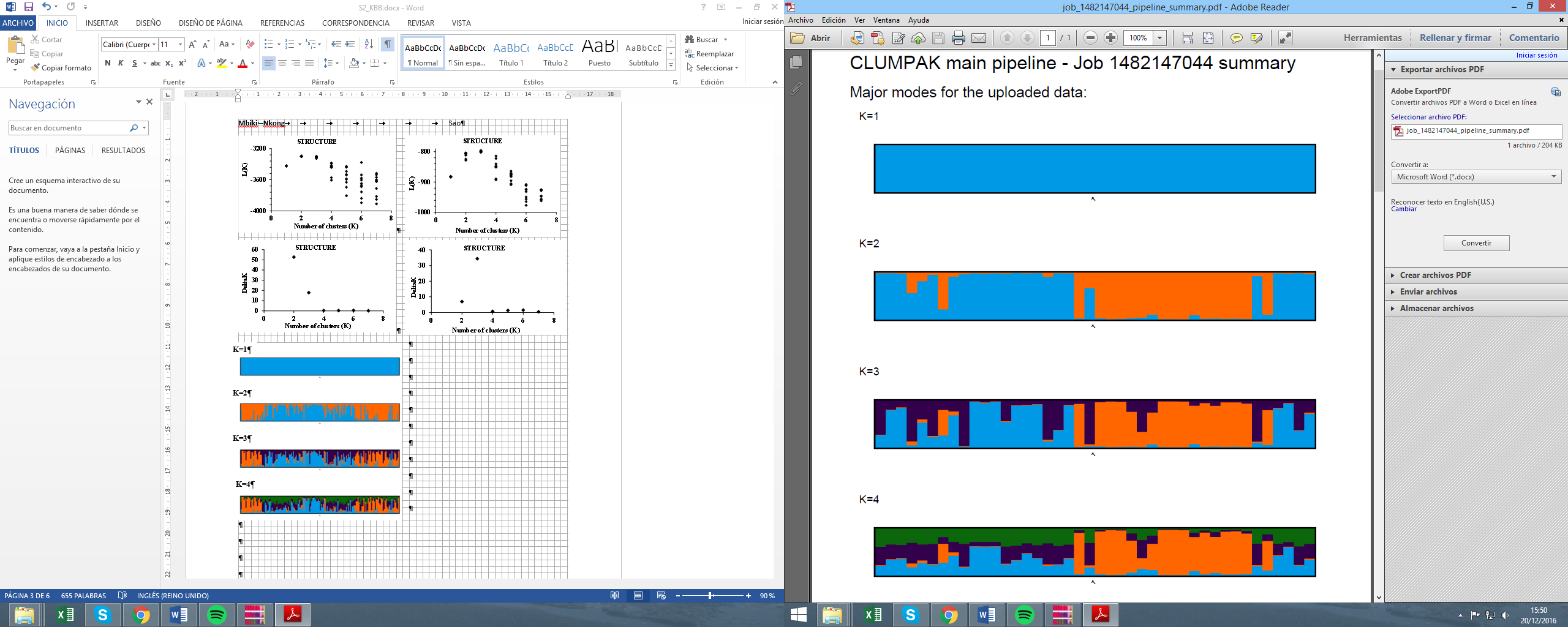


**K=1**

**K=2**

**K=3**

**K=4**

**c) Yasuní, *K*=1**

**d) Ituberá, *K=1***

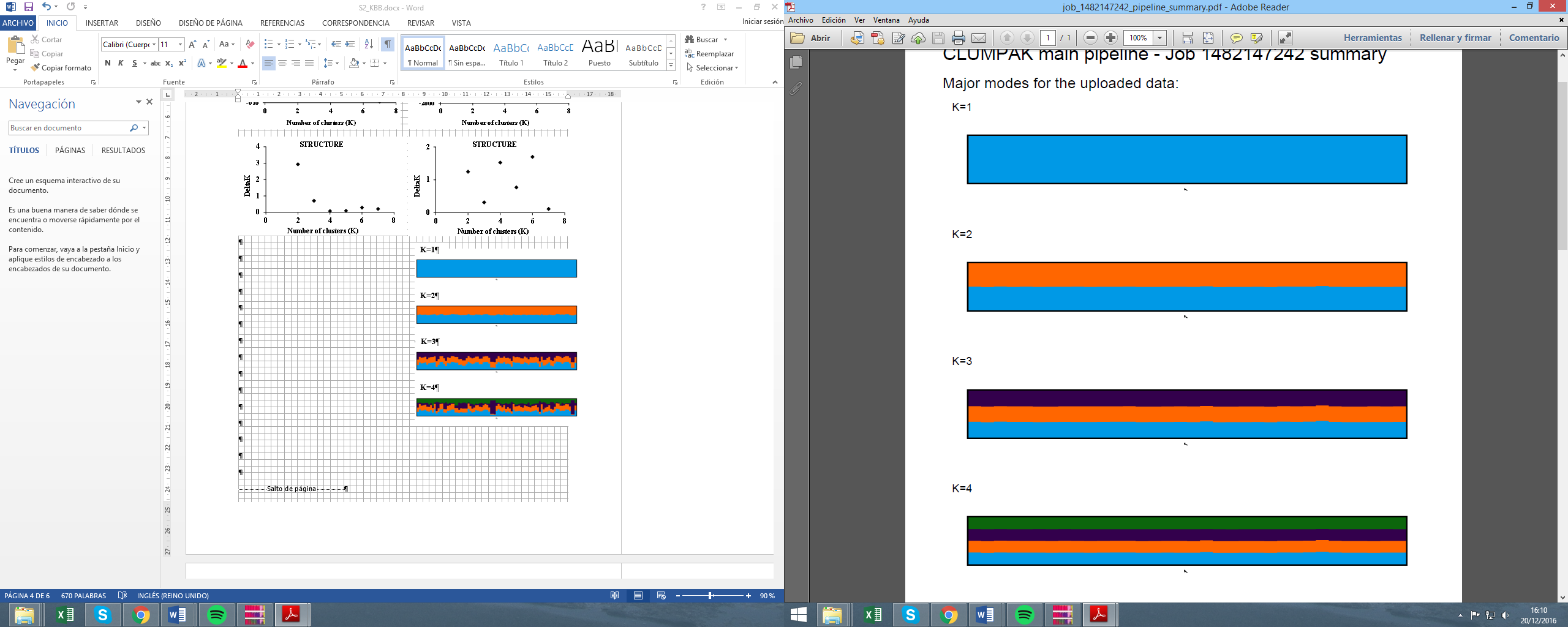


**K=1**

**K=2**

**K=3**

**K=4**


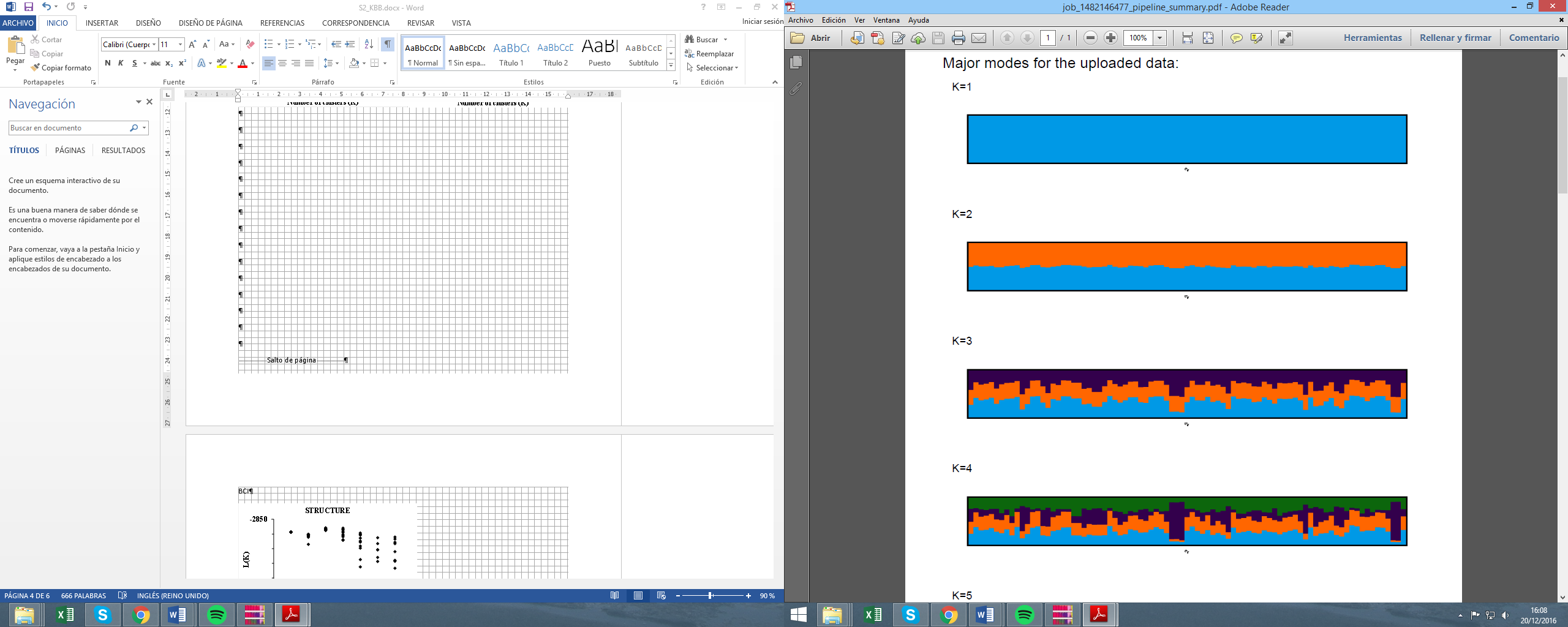


**K=1**

**K=2**

**K=3**

**K=4**

**e) BCI, *K*=3**

**d) Paracou, *K*=1**

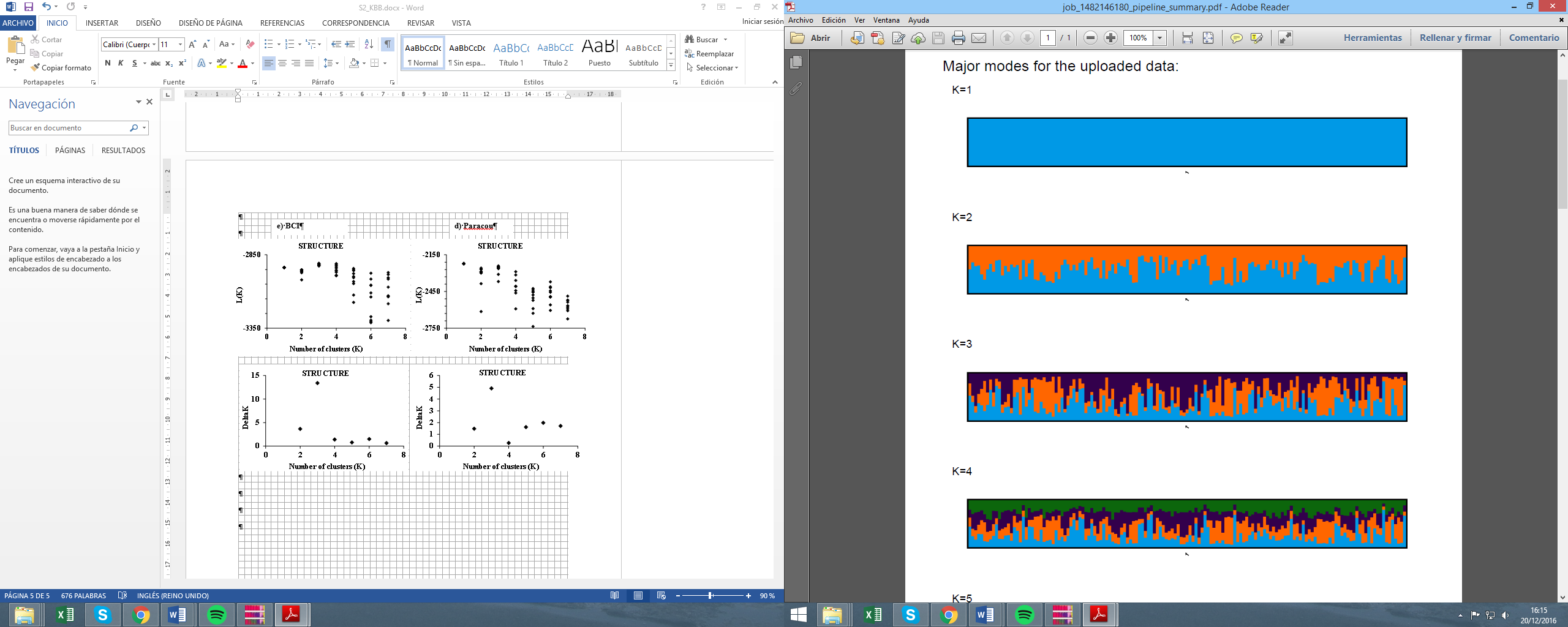


**K=1**

**K=2**

**K=3**

**K=4**


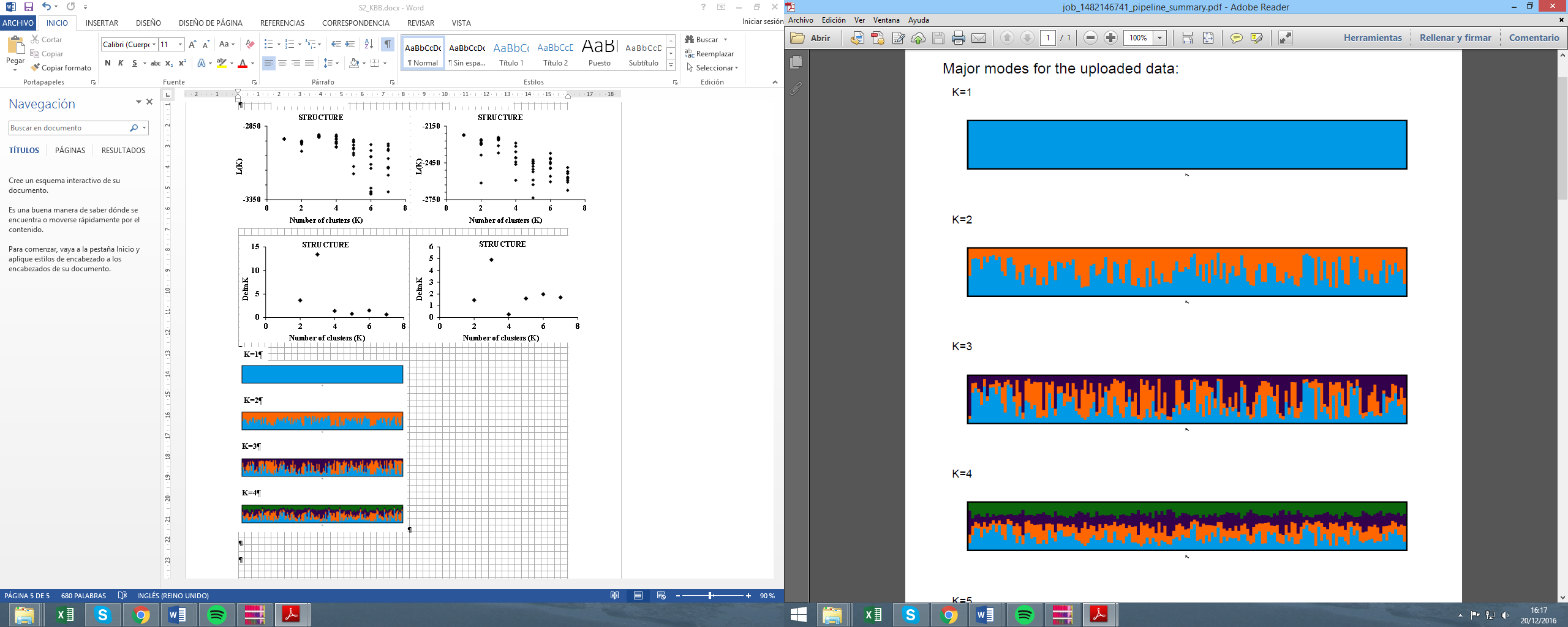


**K=3**

**K=4**

**K=2**

**K=1**

**Comparison of the codominant and recessive marker models in STRUCTURE**

Table S2.1. Comparison of the codominant and recessive marker models in STRUCTURE analyses. *K*, number of clusters under the codominant marker model, *K(null)*, number of clusters under the recessive marker model which accounts for null alleles. *r* *Q*_GP1_ - *r* *Q*_GP3_, Pearson’s *r* correlation coefficients between ancestry proportions for each gene pool between the codominant and recessive allele models.

| Population | *K* | *K(null)* | *r* *Q*_GP1_ | *r Q*_GP2_ | *r* *Q*_GP3_ |
| --- | --- | --- | --- | --- | --- |
| **Neotropics** |  |  |  |  |  |
| BCI | 3 | 3 | 0.988 | 0.968 | 0.989 |
| Yasuní | 1 | 1 | - | - | - |
| Paracou | 1 | 1 | - | - | - |
| Ituberá | 1 | 1 | - | - | - |
| **Africa** |  |  |  |  |  |
| São Tomé | 3 | 3 | 0.938 | 0.992 | 0.973 |
| Nkong Mekak | 2 | 3 | 0.993 | 0.993 | - |
| Mbikiliki | 2 | 3 | 0.988 | 0.988 | - |

**TESS analysis and comparison with the codominant marker model in STRUCTURE**

A Bayesian clustering analysis of individual multilocus genotypes was performed with the program TESS version 2.3.1 [1], which can include the individuals’ spatial position as prior information. We used an admixture model based on trend surfaces and on a conditional autoregressive model (CAR) where each individual’s multilocus genotype is composed of fractions that originate in up to *K* different potentially unobserved source populations or clusters. The degree of trend surface in the model determines whether spatial information is included (0, no spatial information; ≤1, spatial information) and the spatial interaction parameter *Ψ* determines the intensity of spatial autocorrelation. We tested three different models: model 1 with trend degree = 0 and *Ψ* = 0; model 2 with trend degree = 1 and *Ψ* = 0.6; and model 3 with trend degree = 1 and *Ψ* = 1, and used default options for the other parameters (see [2])[2]. Model 1 without spatial prior is equivalent to STRUCTURE, as used in our work (see manuscript). Each model was run for 100,000 MCMC steps, including a burn-in of 20,000 steps, and we repeated the analysis ten times from *K* = 2 to *K* =7. The number of clusters *K* that best described the data was based on the model’s log-likelihood, the deviance information criterion (DIC), and ∆*K* [3–5]. The choice of the *K* that best explained the data was not trivial in TESS, and we systematically obtained a larger *K* than in STRUCTURE (Figures S2.1 and S2.2). In Table S2.1, we illustrate the correlation of cluster membership in STRUCTURE and TESS, choosing *K* based on the STRUCTURE analysis.

Table S2.2. Pearson correlations between ancestry coefficients inferred by STRUCTURE for the best run and *K* (K > 1) and ancestry coefficients of the three models tested in TESS for the best run (choice of *K* based on STRUCTURE analysis). M1: model 1 in TESS, M2: model 2 in TESS; M3: model 3 in TESS. GP1, GP2, GP3: the different GPs identified by the Bayesian clustering. Significance values refer to significance of Pearson correlation tests after Bonferroni correction: ns, not significant; ***, P≤0.001; **, P≤0.01; *, P≤0.05.

|  | GP1 | | |  | | GP2 | | | |  | GP3 | | |
| --- | --- | --- | --- | --- | --- | --- | --- | --- | --- | --- | --- | --- | --- |
| Population | M1 | M2 | M3 | |  | | M1 | M2 | M3 |  | M1 | M2 | M3 |
| Mbikiliki and Nkong Mekak | 0.97^***^ | 0.96^***^ | 0.95^***^ | |  | | 0.97^***^ | 0.96^***^ | 0.95^***^ |  | - | - | - |
| São Tomé | 0.68^***^ | 0.63^***^ | 0.35^ns^ | |  | | 0.99^***^ | 0.92^***^ | 0.88^***^ |  | 0.85^***^ | 0.81^***^ | 0.64^***^ |
| BCI | -0.25^ns^ | -0.19^ns^ | -0.21^ns^ | |  | | -0.53^***^ | 0.03^ns^ | -0.01^ns^ |  | -0.21^ns^ | 0.74^***^ | 0.71^***^ |

Figure S2.2. Illustration of different criteria for the choice of the best number of genetic clusters *K* in TESS analyses for the Cameroonian Mbikiliki and Nkong Mekak populations. In STRUCTURE (*see Fig. S2.1)*, *K=*2 was supported based on model log likelihood (L(K)) and Delta K (∆*K).* In TESS the deviance information criterion (DIC) and the model log likelihood indicated increasing support for increasing *K* values, and the ∆*K* criterion was undefined for *K*=2, because TESS does not run with *K*=1.

**Literature cited**

1. Chen C, Durand E, Forbes F, Fran O. Bayesian clustering algorithms ascertaining spatial population structure: a new computer program and a comparison study. Mol Ecol Notes. 2007;7: 747–756. doi:10.1111/j.1471-8286.2007.01769.x

2. Durand E, Jay F, Gaggiotti OE, François O. Spatial inference of admixture proportions and secondary contact zones. Mol Biol Evol. 2009;26: 1963–1973. doi:10.1093/molbev/msp106

3. Evanno G, Regnaut S, Goudet J. Detecting the number of clusters of individuals using the software STRUCTURE: a simulation study. Mol Ecol. 2005;14: 2611–2620. doi:10.1111/j.1365-294X.2005.02553.x

4. Durand E, Chen C, François O. Tess version 2.3 - Reference Manual. 2009. Available from: http://membres-timc.imag.fr/Olivier.Francois/manual.pdf

5. Pritchard JK, Wen X, Falush D. Documentation for structure software: Version 2.3. 2010. Available from: http://pritchardlab.stanford.edu/structure_software/release_versions/v2.3.4/structure_doc.pdf
