# Supplementary material for: Altitudinal gradients, biogeographic history and microhabitat adaptation affect fine-scale spatial genetic structure in African and Neotropical populations of an ancient tropical tree species
Source: PLoS One. 2017 Aug 3;12(8):e0182515. doi: 10.1371/journal.pone.0182515 (PMC5542443; doi:10.1371/journal.pone.0182515)
Supplement: S3 File — (DOCX) [file pone.0182515.s003.docx]

**S3 File. Evolutionary relationships among plastid DNA haplotypes**

A haplotype network for *psbA- trnH* sequences was created in TCS [1] using statistical parsimony based on a haplotype distance matrix where distance between two haplotypes was defined as the number of genetic differences (nucleotide differences or insertion/deletion polymorphisms).

Fig S3.1. Haplotype network in *Symphonia globulifera* and geographic distribution of haplotypes. Chart size increases with sample size (from 1 to 67 individuals). Haplotype numbers and colors correspond to those of Figs. 2 and 3 in the manuscript. Each line corresponds to one mutation and small white circles indicate non-observed haplotypes.


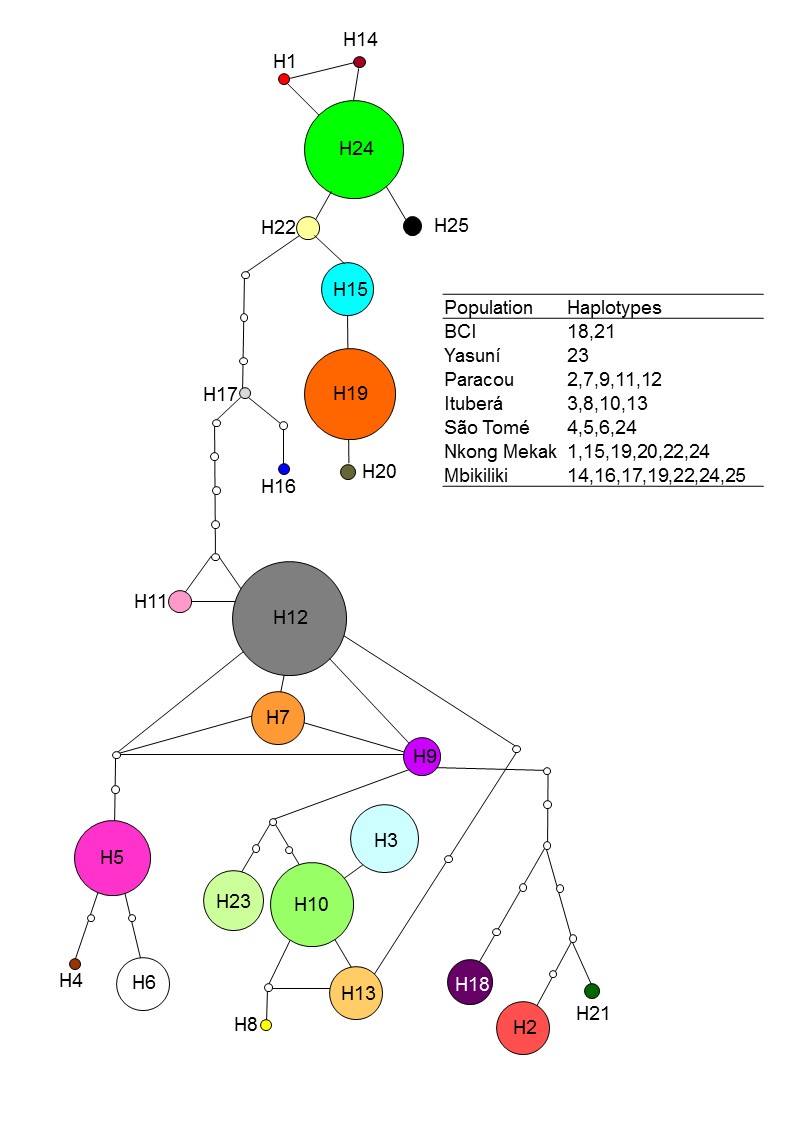


Table S3.1. Plastid DNA haplotype definition in *Symphonia globulifera* based on sequences of the *psbA-trnH* intergenic spacer region. *n*, sample size; column headings indicate positions of single nucleotide polymorphisms, insertion-deletion polymorphisms, microsatellites and inversions.

|  | Polymorphic positions in *psbA-trnH* | | | | | | | | | | | | | | | | | | | | | | | | | | | | |
| --- | --- | --- | --- | --- | --- | --- | --- | --- | --- | --- | --- | --- | --- | --- | --- | --- | --- | --- | --- | --- | --- | --- | --- | --- | --- | --- | --- | --- | --- |
| Hap | n | 70 | 170 | 226 | 248-253 | 265 | 313-314 | 320 | 328-330 | 352 | 355-361 | 411 | 413-425 | 431-444 | 445 | 446-451 | 453 | 483 | 505 | 509 | 511-519 | 520 | 534 | 538-561 | 593 | 614 | 616 | 620-649 | 653 |
| H1 | 1 | ? | T | T | TAAGAA | A | TA | T | TTT | T | TTTTTAC | - | CTCATTTTTCTTT | TTTTTTTTTTT--- | G | TTTTTA | T | T | T | T | - | A | A | ATAA-------A------------G | A | - | G | A----------------------------- | C |
| H2 | 9 | T | T | - | - | A | AT | G | AAA | T | - | A | CTCATTTTTCTTT | TTTTTTTTT----- | T | - | T | G | C | T | - | A | C | ATAA-------AAAATAAA-----G | A | T | G | A----------------------------- | G |
| H3 | 11 | T | G | T | TAAGAA | A | AT | G | AAA | T | - | A | CTCATTTTTCTTT | TTTTTTTTT----- | T | - | T | T | A | T | TTAAATTTG | A | C | ATAA-------AGAATAAA-----G | A | T | A | A----------------------------- | G |
| H4 | 1 | T | T | T | TAAGAA | A | AT | G | TTT | T | - | - | - | TTTTTTTTTTTT-- | T | -----A | T | T | A | T | - | A | C | ATAA-------AGAATAAA-----G | A | T | G | A----------------------------- | G |
| H5 | 27 | T | T | T | TAAGAA | A | AT | G | TTT | T | - | - | - | TTTTTTTTTTTTT- | T | - | T | T | A | T | - | A | C | ATAA-------AGAATAAA-----G | A | T | G | A----------------------------- | G |
| H6 | 9 | T | T | T | TAAGAA | A | AT | G | TTT | T | - | - | - | TTTTTTTTTTTTTT | T | - | G | T | A | T | - | A | C | ATAA-------AGAATAAA-----G | A | T | G | A----------------------------- | G |
| H7 | 9 | T | T | T | TAAGAA | A | AT | G | TTT | T | - | A | CTCATTTTTCTTT | TTTTTTT------- | T | - | T | T | A | T | - | A | C | ATAA-------AGAATAAA-----G | A | T | G | A----------------------------- | G |
| H8 | 1 | T | G | T | TAAGAA | A | AT | G | TTT | T | - | A | CTCATTTTTCTTT | TTTTTTTT------ | T | - | T | T | A | T | TTAAATTTG | A | C | ATAA-------AGAATAAA-----G | C | T | A | A----------------------------- | G |
| H9 | 6 | T | T | T | TAAGAA | A | AT | G | TTT | T | - | A | CTCATTTTTCTTT | TTTTTTTTT----- | T | - | T | T | A | T | - | A | C | ATAA-------AGAATAAA-----G | A | T | G | A----------------------------- | G |
| H10 | 29 | T | G | T | TAAGAA | A | AT | G | TTT | T | - | A | CTCATTTTTCTTT | TTTTTTTTT----- | T | - | T | T | A | T | TTAAATTTG | A | C | ATAA-------AGAATAAA-----G | A | T | A | A----------------------------- | G |
| H11 | 5 | T | T | T | TAAGAA | A | AT | G | TTT | T | - | A | CTCATTTTTCTTT | TTTTTTTTTT---- | T | - | T | T | A | T | - | A | C | ATAA-------AAAATAAA-----G | A | T | G | A----------------------------- | G |
| H12 | 67 | T | T | T | TAAGAA | A | AT | G | TTT | T | - | A | CTCATTTTTCTTT | TTTTTTTTTT---- | T | - | T | T | A | T | - | A | C | ATAA-------AGAATAAA-----G | A | T | G | A----------------------------- | G |
| H13 | 9 | T | G | T | TAAGAA | A | AT | G | TTT | T | - | A | CTCATTTTTCTTT | TTTTTTTTTT---- | T | - | T | T | A | T | TTAAATTTG | A | C | ATAA-------AGAATAAA-----G | A | T | A | A----------------------------- | G |
| H14 | 1 | A | T | T | TAAGAA | A | TA | T | TTT | T | TTTTTAC | - | CTCATTTTTCTTT | TTTTTTTTTTT--- | G | TTTTTA | T | T | T | T | - | A | A | ATAA-------A------------G | A | T | G | A----------------------------- | C |
| H15 | 9 | T | T | T | TAAGAA | C | TA | T | TTT | T | TTTTTAC | - | CTCATTTTTCTTT | TTTTTTTTTT---- | G | TTTTTA | T | T | T | T | - | A | A | ATAA-------A------------G | A | T | G | A----------------------------- | C |
| H16 | 1 | ? | ? | ? | ? | A | TA | T | TTT | T | - | A | CTCATTTTTCTTT | TTTTTTTTT----- | G | TTTTTA | T | A | A | T | - | A | A | ATAA-------A------------G | A | T | G | A----------------------------- | G |
| H17 | 1 | T | T | T | TAAGAA | A | TA | T | TTT | T | - | A | CTCATTTTTCTTT | TTTTTTTTTT---- | G | TTTTTA | T | T | A | T | - | A | A | ATAA-------A------------G | A | T | G | A----------------------------- | G |
| H18 | 8 | T | T | - | TAAGAA | A | AT | G | TTT | G | - | A | CTCATTTTTCTTT | TTTTTTTTT----- | T | - | T | T | C | T | - | T | C | A-------AAAAAAATAAA-----G | A | T | G | A-------TTATTCTTTTATTTTAGTGAA | G |
| H19 | 32 | T | T | T | TAAGAA | C | TA | T | TTT | T | TTTTTAC | - | CTCATTTTTCTTT | TTTTTTTTT----- | G | TTTTTA | T | T | T | T | - | A | A | ATAA-------A------------G | A | T | G | A----------------------------- | C |
| H20 | 2 | T | T | T | TAAGAA | C | TA | T | TTT | T | TTTTTAC | - | CTCATTTTTCTTT | TTTTTTTTT----- | G | TTTTT- | T | T | T | T | - | A | A | ATAA-------A------------G | A | T | G | A----------------------------- | C |
| H21 | 2 | T | T | - | TAAGAA | A | AT | G | TTT | T | - | A | CTCATTTTTCTTT | TTTTTTTTTTT--- | T | - | T | G | C | T | - | A | C | ATAA-------AAAATAAA-----G | A | T | G | A----------------------------- | G |
| H22 | 5 | T | T | T | TAAGAA | A | TA | T | TTT | T | TTTTTAC | - | CTCATTTTTCTTT | TTTTTTTTTT---- | G | TTTTTA | T | T | T | T | - | A | A | ATAA-------A------------G | A | T | G | A----------------------------- | C |
| H23 | 10 | T | T | T | TAAGAA | A | AT | G | TTT | T | - | A | CTCATTTTTCTTT | TTTTTTTTT----- | T | -----A | T | T | A | A | - | A | C | ATAA-------AGAATAAA-----G | A | T | A | A----------------------------- | G |
| H24 | 44 | T | T | T | TAAGAA | A | TA | T | TTT | T | TTTTTAC | - | CTCATTTTTCTTT | TTTTTTTTTTT--- | G | TTTTTA | T | T | T | T | - | A | A | ATAA-------A------------G | A | T | G | A----------------------------- | C |
| H25 | 4 | T | T | T | TAAGAA | A | TA | T | TTT | T | TTTTTAC | - | CTCATTTTTCTTT | TTTTTTTTTTT--- | G | TTTTT- | T | T | T | T | - | A | A | ATAA-------A------------G | A | T | G | A----------------------------- | C |

Table S3.2 *Psba-trnH* sequence data set in *Symphonia globulifera* with Genbank accession numbers. In Paracou, the *S. globulifera* morphotype is given in the Population field: *S.glo* for the common morphotype, and *S.sp1* for the alternative morphotype.

| Individual ID | Population | Latitude | Longitude | accession number | Haplotype |
| --- | --- | --- | --- | --- | --- |
| Sg_EU841720 | BCI | - | - | EU841720 | H18 |
| Sg_EU841721 | BCI | - | - | EU841721 | H18 |
| Sg_EU841722 | BCI | - | - | EU841722 | H18 |
| Sg_EU841723 | BCI | - | - | EU841723 | H18 |
| Sg_EU841724 | BCI | - | - | EU841724 | H18 |
| Sg_EU841725 | BCI | - | - | EU841725 | H18 |
| Sg_EU841726 | BCI | - | - | EU841726 | H21 |
| Sg_EU841727 | BCI | - | - | EU841727 | H18 |
| Sg_EU841728 | BCI | - | - | EU841728 | H21 |
| Sg_GQ982376 | BCI | - | - | GQ982376 | H18 |
| KH01 | Ituberá | -13.78642 | -39.17686 | KX572535 | H10 |
| KH02 | Ituberá | -13.78650 | -39.17688 | KX572536 | H10 |
| KH03 | Ituberá | -13.78848 | -39.17795 | KX572537 | H13 |
| KH05 | Ituberá | -13.79027 | -39.17832 | KX572538 | H10 |
| KH07 | Ituberá | -13.79532 | -39.17937 | KX572539 | H10 |
| KH08 | Ituberá | -13.79551 | -39.17949 | KX572540 | H10 |
| KH10 | Ituberá | -13.78683 | -39.17719 | KX572541 | H3 |
| KH12 | Ituberá | -13.78989 | -39.17823 | KX572542 | H10 |
| KH13 | Ituberá | -13.79218 | -39.17887 | KX572543 | H13 |
| KH15 | Ituberá | -13.79434 | -39.17888 | KX572544 | H13 |
| KH17 | Ituberá | -13.79577 | -39.17968 | KX572545 | H10 |
| KH19 | Ituberá | -13.79634 | -39.18025 | KX572546 | H8 |
| KH23 | Ituberá | -13.79887 | -39.18072 | KX572547 | H10 |
| KH27 | Ituberá | -13.79758 | -39.18064 | KX572548 | H13 |
| KH29 | Ituberá | -13.79864 | -39.18091 | KX572549 | H10 |
| KH31 | Ituberá | -13.79416 | -39.17895 | KX572550 | H13 |
| KH32 | Ituberá | -13.79126 | -39.17854 | KX572551 | H10 |
| KH34 | Ituberá | -13.79704 | -39.18040 | KX572552 | H10 |
| KH36 | Ituberá | -13.79644 | -39.18138 | KX572553 | H3 |
| KH37 | Ituberá | -13.79613 | -39.18152 | KX572554 | H3 |
| KH38 | Ituberá | -13.79608 | -39.18152 | KX572555 | H10 |
| KH40 | Ituberá | -13.79526 | -39.18167 | KX572556 | H10 |
| KH42 | Ituberá | -13.79571 | -39.18149 | KX572557 | H10 |
| KH43 | Ituberá | -13.79547 | -39.18148 | KX572558 | H10 |
| KH45 | Ituberá | -13.78970 | -39.17811 | KX572559 | H3 |
| KH48 | Ituberá | -13.79680 | -39.18049 | KX572560 | H10 |
| KH50 | Ituberá | -13.78664 | -39.17695 | KX572561 | H10 |
| KH53 | Ituberá | -13.79434 | -39.17892 | KX572562 | H10 |
| KH55 | Ituberá | -13.79395 | -39.18010 | KX572563 | H3 |
| KH56 | Ituberá | -13.79393 | -39.18078 | KX572564 | H10 |
| KH58 | Ituberá | -13.79353 | -39.18000 | KX572565 | H13 |
| KH60 | Ituberá | -13.79369 | -39.17946 | KX572566 | H10 |
| KH61 | Ituberá | -13.79372 | -39.17940 | KX572567 | H13 |
| KH62 | Ituberá | -13.79385 | -39.17906 | KX572568 | H3 |
| KH64 | Ituberá | -13.79381 | -39.17911 | KX572569 | H10 |
| KH66 | Ituberá | -13.79384 | -39.17919 | KX572570 | H13 |
| KH68 | Ituberá | -13.79385 | -39.17930 | KX572571 | H10 |
| KH70 | Ituberá | -13.79388 | -39.17930 | KX572572 | H10 |
| KH71 | Ituberá | -13.79389 | -39.17927 | KX572573 | H10 |
| KH72 | Ituberá | -13.79355 | -39.17973 | KX572574 | H10 |
| KH74 | Ituberá | -13.79022 | -39.17833 | KX572575 | H3 |
| KH76 | Ituberá | -13.79825 | -39.18085 | KX572576 | H3 |
| KH77 | Ituberá | -13.79836 | -39.18085 | KX572577 | H13 |
| KH78 | Ituberá | -13.79753 | -39.18065 | KX572578 | H3 |
| KH82 | Ituberá | -13.79680 | -39.18141 | KX572579 | H10 |
| KH84 | Ituberá | -13.79620 | -39.18144 | KX572580 | H3 |
| KH85 | Ituberá | -13.79589 | -39.18156 | KX572581 | H3 |
| KH87 | Ituberá | -13.79579 | -39.18143 | KX572582 | H10 |
| KH89 | Ituberá | -13.79564 | -39.18139 | KX572583 | H10 |
| KH90 | Ituberá | -13.79507 | -39.18174 | KX572584 | H10 |
| MH2503 | Mbikiliki | 3.18985 | 10.52720 | KX572632 | H19 |
| MH2505 | Mbikiliki | 3.18967 | 10.52713 | KX572633 | H19 |
| MH2508 | Mbikiliki | 3.18871 | 10.52741 | KX572634 | H19 |
| MH2509 | Mbikiliki | 3.18865 | 10.52721 | KX572635 | H16 |
| MH2511 | Mbikiliki | 3.18882 | 10.52682 | KX572636 | H19 |
| MH2514 | Mbikiliki | 3.18872 | 10.52649 | KX572637 | H19 |
| MH2515 | Mbikiliki | 3.18808 | 10.52629 | KX572638 | H19 |
| MH2518 | Mbikiliki | 3.18807 | 10.52617 | KX572639 | H19 |
| MH2521 | Mbikiliki | 3.18790 | 10.52588 | KX572640 | H19 |
| MH2527 | Mbikiliki | 3.18599 | 10.52464 | KX572641 | H19 |
| MH2531 | Mbikiliki | 3.18907 | 10.52651 | KX572642 | H19 |
| MH2537 | Mbikiliki | 3.19142 | 10.52547 | KX572643 | H19 |
| MH2543 | Mbikiliki | 3.19458 | 10.52202 | KX572644 | H24 |
| MH2552 | Mbikiliki | 3.19636 | 10.52143 | KX572645 | H24 |
| MH2557 | Mbikiliki | 3.19688 | 10.52293 | KX572646 | H24 |
| MH2560 | Mbikiliki | 3.19710 | 10.52332 | KX572647 | H24 |
| MH2564 | Mbikiliki | 3.19700 | 10.52405 | KX572648 | H24 |
| MH2567 | Mbikiliki | 3.19711 | 10.52392 | KX572649 | H24 |
| MH2569 | Mbikiliki | 3.19741 | 10.52422 | KX572650 | H24 |
| MH2571 | Mbikiliki | 3.19746 | 10.52442 | KX572651 | H24 |
| MH2572 | Mbikiliki | 3.19672 | 10.52449 | KX572652 | H24 |
| MH2575 | Mbikiliki | 3.19611 | 10.52263 | KX572653 | H24 |
| MH2579 | Mbikiliki | 3.19515 | 10.52206 | KX572654 | H24 |
| MH2582 | Mbikiliki | 3.19504 | 10.52190 | KX572655 | H24 |
| MH2586 | Mbikiliki | 3.19105 | 10.52591 | KX572656 | H24 |
| MH2587 | Mbikiliki | 3.19093 | 10.52631 | KX572657 | H22 |
| MH2590 | Mbikiliki | 3.18585 | 10.52963 | KX572658 | H24 |
| MH2596 | Mbikiliki | 3.18350 | 10.53468 | KX572659 | H24 |
| MH2610 | Mbikiliki | 3.18510 | 10.53664 | KX572660 | H24 |
| MH2623 | Mbikiliki | 3.18656 | 10.53873 | KX572661 | H24 |
| MH2626 | Mbikiliki | 3.18659 | 10.53934 | KX572662 | H24 |
| MH2629 | Mbikiliki | 3.18675 | 10.53956 | KX572663 | H24 |
| MH2630 | Mbikiliki | 3.18701 | 10.53958 | KX572664 | H24 |
| MH2632 | Mbikiliki | 3.18725 | 10.53961 | KX572665 | H24 |
| MH2636 | Mbikiliki | 3.18695 | 10.53996 | KX572666 | H24 |
| MH2640 | Mbikiliki | 3.18721 | 10.54044 | KX572667 | H24 |
| MH2646 | Mbikiliki | 3.18669 | 10.54080 | KX572668 | H24 |
| MH2649 | Mbikiliki | 3.18672 | 10.54019 | KX572669 | H24 |
| MH2651 | Mbikiliki | 3.18665 | 10.54010 | KX572670 | H24 |
| OH1263 | Mbikiliki | 3.17730 | 10.54070 | JQ996311 | H25 |
| OH1264 | Mbikiliki | 3.18510 | 10.53690 | JQ996312 | H25 |
| OH1265 | Mbikiliki | 3.18850 | 10.53800 | JQ996313 | H14 |
| OH1290 | Mbikiliki | 3.18650 | 10.53870 | JQ996315 | H24 |
| OH1291 | Mbikiliki | 3.19310 | 10.53640 | JQ996316 | H24 |
| OH1292 | Mbikiliki | 3.18110 | 10.53130 | JQ996317 | H25 |
| OH1293 | Mbikiliki | 3.17060 | 10.52940 | JQ996318 | H25 |
| OH1294 | Mbikiliki | 3.18230 | 10.52950 | JQ996319 | H17 |
| OH1295 | Mbikiliki | 3.18410 | 10.52880 | KX572671 | H24 |
| OH1299 | Mbikiliki | 3.18490 | 10.52750 | JQ996320 | H24 |
| OH1300 | Mbikiliki | 3.18390 | 10.52640 | JQ996321 | H24 |
| MH1621 | Nkong Mekak | 2.79150 | 10.53470 | KX572585 | H20 |
| MH1630 | Nkong Mekak | 2.78960 | 10.53550 | JQ996269 | H20 |
| MH2374 | Nkong Mekak | 2.74938 | 10.53954 | KX660691 | H24 |
| MH2381 | Nkong Mekak | 2.76667 | 10.53692 | KX572586 | H24 |
| MH2385 | Nkong Mekak | 2.76624 | 10.53409 | KX572587 | H24 |
| MH2386 | Nkong Mekak | 2.76638 | 10.53390 | KX572588 | H24 |
| MH2395 | Nkong Mekak | 2.76437 | 10.53282 | KX572589 | H24 |
| MH2397 | Nkong Mekak | 2.76394 | 10.53278 | KX572590 | H24 |
| MH2398 | Nkong Mekak | 2.76392 | 10.53282 | KX572591 | H24 |
| MH2400 | Nkong Mekak | 2.76359 | 10.53270 | KX572592 | H24 |
| MH2401 | Nkong Mekak | 2.76341 | 10.53282 | KX572593 | H24 |
| MH2403 | Nkong Mekak | 2.76332 | 10.53274 | KX572594 | H24 |
| MH2405 | Nkong Mekak | 2.76314 | 10.53263 | KX572595 | H24 |
| MH2406 | Nkong Mekak | 2.76310 | 10.53264 | KX572596 | H22 |
| MH2407 | Nkong Mekak | 2.76298 | 10.53281 | KX572597 | H1 |
| MH2409 | Nkong Mekak | 2.76317 | 10.53212 | KX572598 | H22 |
| MH2412 | Nkong Mekak | 2.76289 | 10.53184 | KX572599 | H24 |
| MH2415 | Nkong Mekak | 2.76254 | 10.53166 | KX572600 | H22 |
| MH2416 | Nkong Mekak | 2.76254 | 10.53166 | KX572601 | H22 |
| MH2418 | Nkong Mekak | 2.76222 | 10.53147 | KX572602 | H19 |
| MH2419 | Nkong Mekak | 2.76204 | 10.53136 | KX572603 | H19 |
| MH2420 | Nkong Mekak | 2.76107 | 10.53148 | KX572604 | H15 |
| MH2424 | Nkong Mekak | 2.75923 | 10.53160 | KX572605 | H15 |
| MH2425 | Nkong Mekak | 2.75935 | 10.53166 | KX572606 | H15 |
| MH2426 | Nkong Mekak | 2.75790 | 10.53283 | KX572607 | H15 |
| MH2428 | Nkong Mekak | 2.75598 | 10.53256 | KX572608 | H15 |
| MH2430 | Nkong Mekak | 2.75392 | 10.53293 | KX572609 | H19 |
| MH2438 | Nkong Mekak | 2.76332 | 10.54181 | KX572610 | H15 |
| MH2439 | Nkong Mekak | 2.76334 | 10.54186 | KX572611 | H19 |
| MH2441 | Nkong Mekak | 2.76315 | 10.54182 | KX572612 | H15 |
| MH2445 | Nkong Mekak | 2.76309 | 10.54193 | KX572613 | H15 |
| MH2447 | Nkong Mekak | 2.76269 | 10.54228 | KX572614 | H19 |
| MH2448 | Nkong Mekak | 2.76276 | 10.54226 | KX572615 | H19 |
| MH2450 | Nkong Mekak | 2.76273 | 10.54239 | KX572616 | H19 |
| MH2453 | Nkong Mekak | 2.76263 | 10.54297 | KX572617 | H19 |
| MH2458 | Nkong Mekak | 2.76143 | 10.54365 | KX572618 | H19 |
| MH2461 | Nkong Mekak | 2.76090 | 10.54445 | KX572619 | H19 |
| MH2462 | Nkong Mekak | 2.76000 | 10.54500 | KX572620 | H19 |
| MH2465 | Nkong Mekak | 2.76004 | 10.54494 | KX572621 | H19 |
| MH2466 | Nkong Mekak | 2.75970 | 10.54506 | KX572622 | H19 |
| MH2472 | Nkong Mekak | 2.75895 | 10.54532 | KX572623 | H19 |
| MH2478 | Nkong Mekak | 2.75865 | 10.54514 | KX572624 | H19 |
| MH2482 | Nkong Mekak | 2.75754 | 10.54480 | KX572625 | H19 |
| MH2483 | Nkong Mekak | 2.75737 | 10.54488 | KX572626 | H19 |
| MH2486 | Nkong Mekak | 2.75672 | 10.54470 | KX572627 | H15 |
| MH2487 | Nkong Mekak | 2.75672 | 10.54476 | KX572628 | H19 |
| MH2489 | Nkong Mekak | 2.75649 | 10.54468 | KX572629 | H19 |
| MH2495 | Nkong Mekak | 2.75585 | 10.54527 | KX572630 | H19 |
| MH2496 | Nkong Mekak | 2.75595 | 10.54535 | KX572631 | H19 |
| 1_1_231 | Paracou S.glo | 5.27146 | -52.93880 | KX572474 | H12 |
| 1_1_252 | Paracou S.glo | 5.27151 | -52.93880 | KX572475 | H9 |
| 1_1_255 | Paracou S.glo | 5.27155 | -52.93880 | KX572476 | H9 |
| 1_1_880 | Paracou S.sp1 | 5.27196 | -52.93820 | KX572477 | H9 |
| 1_2_387 | Paracou S.sp1 | 5.27189 | -52.93750 | KX572478 | H12 |
| 1_2_392 | Paracou S.glo | 5.27200 | -52.93760 | KX572479 | H12 |
| 10_1_3300 | Paracou S.sp1 | 5.26011 | -52.92570 | KX572421 | H11 |
| 10_1_746 | Paracou S.sp1 | 5.25981 | -52.92570 | KX572422 | H2 |
| 10_2_948 | Paracou S.glo | 5.26013 | -52.92450 | KX572423 | H12 |
| 10_4_2668 | Paracou S.sp1 | 5.25993 | -52.92490 | KX572424 | H2 |
| 10_4_2947 | Paracou S.glo | 5.25949 | -52.92440 | KX572425 | H12 |
| 10_4_592 | Paracou S.sp1 | 5.25988 | -52.92500 | KX572426 | H2 |
| 11_1_874 | Paracou S.glo | 5.25712 | -52.92920 | KX572427 | H2 |
| 11_2_842 | Paracou S.sp1 | 5.25697 | -52.92800 | KX572428 | H2 |
| 13_1_2849 | Paracou S.glo | 5.27308 | -52.93590 | KX572429 | H7 |
| 13_1_894 | Paracou S.sp1 | 5.27374 | -52.93610 | KX572430 | H12 |
| 13_1_941 | Paracou S.sp1 | 5.27335 | -52.93590 | KX572431 | H12 |
| 13_2_290 | Paracou S.sp1 | 5.27396 | -52.93530 | KX572432 | H12 |
| 13_2_300 | Paracou S.glo | 5.27403 | -52.93530 | KX572433 | H7 |
| 13_2_301 | Paracou S.glo | 5.27406 | -52.93530 | KX572434 | H7 |
| 13_2_304 | Paracou S.glo | 5.27407 | -52.93530 | KX572435 | H7 |
| 13_2_469 | Paracou S.sp1 | 5.27403 | -52.93530 | KX572436 | H12 |
| 13_2_472 | Paracou S.sp1 | 5.27406 | -52.93530 | KX572437 | H12 |
| 13_2_504 | Paracou S.sp1 | 5.27411 | -52.93520 | KX572438 | H12 |
| 13_2_662 | Paracou S.sp1 | 5.27397 | -52.93510 | KX572439 | H12 |
| 13_2_709 | Paracou S.sp1 | 5.27417 | -52.93500 | KX572440 | H12 |
| 13_2_834 | Paracou S.sp1 | 5.27420 | -52.93490 | KX572441 | H12 |
| 13_2_931 | Paracou S.glo | 5.27347 | -52.93445 | KX572442 | H9 |
| 13_3_436 | Paracou S.glo | 5.27190 | -52.93580 | KX572443 | H9 |
| 13_3_538 | Paracou S.glo | 5.27241 | -52.93580 | KX572444 | H12 |
| 13_4_361 | Paracou S.glo | 5.27293 | -52.93510 | KX572445 | H12 |
| 14_1_25 | Paracou S.sp1 | 5.27423 | -52.93330 | KX572446 | H12 |
| 14_1_2830 | Paracou S.sp1 | 5.27438 | -52.93230 | KX572447 | H12 |
| 14_1_587 | Paracou S.glo | 5.27413 | -52.93250 | KX572448 | H12 |
| 14_1_7 | Paracou S.sp1 | 5.27398 | -52.93320 | KX572449 | H12 |
| 14_1_771 | Paracou S.sp1 | 5.27438 | -52.93230 | KX572450 | H12 |
| 14_2_220 | Paracou S.sp1 | 5.27487 | -52.93200 | KX572451 | H12 |
| 14_2_2874 | Paracou S.sp1 | 5.27526 | -52.93160 | KX572452 | H12 |
| 14_2_307 | Paracou S.sp1 | 5.27509 | -52.93190 | KX572453 | H12 |
| 14_2_318 | Paracou S.glo | 5.27492 | -52.93190 | KX572454 | H12 |
| 14_2_459 | Paracou S.glo | 5.27520 | -52.93180 | KX572455 | H12 |
| 14_2_473 | Paracou S.sp1 | 5.27503 | -52.93170 | KX572456 | H12 |
| 14_2_581 | Paracou S.sp1 | 5.27512 | -52.93170 | KX572457 | H12 |
| 14_2_67 | Paracou S.glo | 5.27511 | -52.93230 | KX572458 | H12 |
| 14_2_946 | Paracou S.glo | 5.27489 | -52.93180 | KX572459 | H7 |
| 14_3_263 | Paracou S.sp1 | 5.27376 | -52.93270 | KX572460 | H12 |
| 14_3_445 | Paracou S.sp1 | 5.27396 | -52.93260 | KX572461 | H12 |
| 14_3_663 | Paracou S.sp1 | 5.27365 | -52.93230 | KX572462 | H12 |
| 14_4_770 | Paracou S.glo | 5.27365 | -52.93110 | KX572463 | H12 |
| 15_1_560 | Paracou S.glo | 5.27776 | -52.92770 | KX572464 | H12 |
| 15_1_575 | Paracou S.glo | 5.27799 | -52.92770 | KX572465 | H12 |
| 15_1_637 | Paracou S.glo | 5.27806 | -52.92770 | KX572466 | H12 |
| 15_4_1098 | Paracou S.glo | 5.27720 | -52.92600 | KX572467 | H12 |
| 15_4_655 | Paracou S.glo | 5.27693 | -52.92630 | KX572468 | H7 |
| 15_4_743 | Paracou S.glo | 5.27702 | -52.92630 | KX572469 | H7 |
| 15_4_938 | Paracou S.glo | 5.27697 | -52.92600 | KX572470 | H12 |
| 16_20_42 | Paracou S.glo | 5.25740 | -52.93490 | KX572471 | H11 |
| 16_23_439 | Paracou S.glo | 5.25604 | -52.93580 | KX572472 | H2 |
| 16_3_322 | Paracou S.sp1 | 5.26015 | -52.93700 | KX572473 | H12 |
| 2_1_29 | Paracou S.glo | 5.26863 | -52.93960 | KX572480 | H12 |
| 2_2_413 | Paracou S.sp1 | 5.26927 | -52.93810 | KX572481 | H12 |
| 2_3_200 | Paracou S.glo | 5.26781 | -52.93920 | KX572482 | H7 |
| 2_3_254 | Paracou S.sp1 | 5.26794 | -52.93900 | KX572483 | H9 |
| 2_3_360 | Paracou S.glo | 5.26839 | -52.93900 | KX572484 | H7 |
| 3_1_3165 | Paracou S.sp1 | 5.26911 | -52.93690 | KX572485 | H12 |
| 4_1_3000 | Paracou S.sp1 | 5.27201 | -52.93220 | KX572486 | H12 |
| 4_1_499 | Paracou S.glo | 5.27190 | -52.93210 | KX572487 | H12 |
| 4_4_390 | Paracou S.glo | 5.27061 | -52.93060 | KX572488 | H12 |
| 4_4_693 | Paracou S.glo | 5.27082 | -52.93040 | KX572489 | H12 |
| 5_2_3092 | Paracou S.sp1 | 5.27238 | -52.92780 | KX572490 | H12 |
| 5_3_395 | Paracou S.glo | 5.27144 | -52.92930 | KX572491 | H12 |
| 5_4_626 | Paracou S.glo | 5.27192 | -52.92800 | KX572492 | H12 |
| 5_4_658 | Paracou S.glo | 5.27166 | -52.92790 | KX572493 | H12 |
| 5_4_784 | Paracou S.glo | 5.27174 | -52.92780 | KX572494 | H12 |
| 6_1_21 | Paracou S.sp1 | 5.27571 | -52.92798 | KX572495 | H12 |
| 6_1_2892 | Paracou S.sp1 | 5.27644 | -52.92754 | KX572496 | H12 |
| 6_1_553 | Paracou S.sp1 | 5.27650 | -52.92761 | KX572497 | H12 |
| 6_1_578 | Paracou S.sp1 | 5.27313 | -52.92670 | KX572498 | H12 |
| 6_1_643 | Paracou S.sp1 | 5.27570 | -52.92734 | KX572499 | H12 |
| 6_1_654 | Paracou S.sp1 | 5.27578 | -52.92726 | KX572500 | H12 |
| 6_1_763 | Paracou S.sp1 | 5.27619 | -52.92731 | KX572501 | H12 |
| 6_2_240 | Paracou S.sp1 | 5.27258 | -52.92570 | KX572502 | H12 |
| 6_3_346 | Paracou S.glo | 5.27130 | -52.92650 | KX572503 | H12 |
| 6_3_59 | Paracou S.glo | 5.27150 | -52.92690 | KX572504 | H12 |
| 6_3_610 | Paracou S.glo | 5.27132 | -52.92630 | KX572505 | H12 |
| 6_3_97 | Paracou S.glo | 5.27188 | -52.92690 | KX572506 | H11 |
| 6_4_2864 | Paracou S.sp1 | 5.27174 | -52.92540 | KX572507 | H12 |
| 6_4_2867 | Paracou S.sp1 | 5.27169 | -52.92570 | KX572508 | H11 |
| 7_3_2812 | Paracou S.glo | 5.26696 | -52.93000 | KX572509 | H12 |
| 7_3_907 | Paracou S.sp1 | 5.26768 | -52.92990 | KX572510 | H12 |
| 7_4_299 | Paracou S.glo | 5.26803 | -52.92940 | KX572511 | H12 |
| 8_1_144 | Paracou S.glo | 5.26405 | -52.93150 | KX572512 | H2 |
| 8_1_661 | Paracou S.sp1 | 5.26494 | -52.93110 | KX572513 | H2 |
| 8_3_324 | Paracou S.sp1 | 5.26319 | -52.93100 | KX572514 | H2 |
| 8_3_3289 | Paracou S.sp1 | 5.26362 | -52.93140 | KX572515 | H12 |
| 8_3_3291 | Paracou S.glo | 5.26353 | -52.93130 | KX572516 | H11 |
| FO0001 | São Tomé | 0.29609 | 6.60100 | KX572517 | H5 |
| FO0004 | São Tomé | 0.29610 | 6.60090 | KX572518 | H5 |
| FO0008 | São Tomé | 0.28550 | 6.55900 | KX572519 | H5 |
| FO0011 | São Tomé | 0.28400 | 6.55780 | KX572520 | H5 |
| FO0013 | São Tomé | 0.28380 | 6.55000 | KX572521 | H5 |
| FO0014 | São Tomé | 0.28380 | 6.55000 | KX572522 | H5 |
| FO0018 | São Tomé | 0.29700 | 6.55805 | KX572523 | H5 |
| FO0023 | São Tomé | 0.28390 | 6.55740 | KX572524 | H5 |
| FO0024 | São Tomé | 0.28390 | 6.55740 | KX572525 | H5 |
| FO0028 | São Tomé | 0.28340 | 6.53740 | KX572526 | H5 |
| FO0033 | São Tomé | 0.28350 | 6.55660 | KX572527 | H5 |
| FO0036 | São Tomé | 0.28290 | 6.55490 | KX572528 | H5 |
| FO0043 | São Tomé | 0.28250 | 6.55430 | KX572529 | H5 |
| GID0562 | São Tomé | 0.28060 | 6.58768 | KX572530 | H6 |
| GID0564 | São Tomé | 0.28458 | 6.58668 | KX572531 | H5 |
| GID0565 | São Tomé | 0.28220 | 6.59003 | KX572532 | H6 |
| GID0566 | São Tomé | 0.28024 | 6.59147 | KX572533 | H6 |
| GID0619 | São Tomé | 0.28943 | 6.59554 | KX572534 | H5 |
| OH1504 | São Tomé | 0.28878 | 6.61259 | KX572672 | H6 |
| OH1505 | São Tomé | 0.27126 | 6.57902 | KX572673 | H6 |
| OH1506 | São Tomé | 0.27130 | 6.57904 | KX572674 | H6 |
| OH1507 | São Tomé | 0.27078 | 6.57872 | KX572675 | H6 |
| OH1508 | São Tomé | 0.27085 | 6.57864 | KX572676 | H6 |
| OH1509 | São Tomé | 0.27067 | 6.57805 | KX572677 | H6 |
| OH1511 | São Tomé | 0.26731 | 6.57324 | KX572678 | H5 |
| OH1512 | São Tomé | 0.26627 | 6.57193 | KX572679 | H5 |
| OH1513 | São Tomé | 0.26366 | 6.56796 | KX572680 | H5 |
| OH1515 | São Tomé | 0.26150 | 6.55416 | KX572681 | H4 |
| OH1516 | São Tomé | 0.26414 | 6.55220 | JQ996329 | H5 |
| OH1517 | São Tomé | 0.26486 | 6.55190 | JQ996330 | H5 |
| OH1518 | São Tomé | 0.26612 | 6.54999 | KX572682 | H5 |
| OH1519 | São Tomé | 0.27056 | 6.54607 | KX572683 | H24 |
| OH1520 | São Tomé | 0.27311 | 6.54754 | JQ996331 | H5 |
| OH1521 | São Tomé | 0.27701 | 6.54877 | KX572684 | H5 |
| OH1522 | São Tomé | 0.27857 | 6.54951 | KX572685 | H5 |
| OH1524 | São Tomé | 0.28214 | 6.55276 | KX572686 | H5 |
| OH1526 | São Tomé | 0.28354 | 6.55419 | JQ996332 | H5 |
| OH1527 | São Tomé | 0.28453 | 6.55613 | JQ996333 | H5 |
| Sg_EU841867 | Yasuní | - | - | EU841867 | H23 |
| Sg_EU841868 | Yasuní | - | - | EU841868 | H23 |
| Sg_EU841869 | Yasuní | - | - | EU841869 | H23 |
| Sg_EU841870 | Yasuní | - | - | EU841870 | H23 |
| Sg_EU841871 | Yasuní | - | - | EU841871 | H23 |
| Sg_EU841872 | Yasuní | - | - | EU841872 | H23 |
| Sg_EU841873 | Yasuní | - | - | EU841873 | H23 |
| Sg_EU841874 | Yasuní | - | - | EU841874 | H23 |
| Sg_EU841875 | Yasuní | - | - | EU841875 | H23 |
| Sg_EU841876 | Yasuní | - | - | EU841876 | H23 |

**Literature cited**

1. Clement M, Posada D, Crandall KA. TCS: a computer program to estimate gene genealogies. Mol Ecol. 2000;9: 1657–1659. doi:10.1046/j.1365-294x.2000.01020.x
