# Supplementary material for: Altitudinal gradients, biogeographic history and microhabitat adaptation affect fine-scale spatial genetic structure in African and Neotropical populations of an ancient tropical tree species
Source: PLoS One. 2017 Aug 3;12(8):e0182515. doi: 10.1371/journal.pone.0182515 (PMC5542443; doi:10.1371/journal.pone.0182515)
Supplement: S4 File — (DOCX) [file pone.0182515.s004.docx]

**S4 File. Genetic diversity and fine-scale spatial genetic structure statistics in *Symphonia globulifera* based on different groups of SSRs.**

Table S4.1. Genetic diversity and fine-scale spatial genetic structure statistics in *Symphonia globulifera* in a subset of loci corresponding to the three nuclear SSRs used in Paracou [1]. n, sample size for SSR data; *A*, mean number of alleles per locus; *A*_R_, allelic richness or number of alleles expected in a sample of 34 individuals and standard deviation; *H*_E_, expected heterozygosity; *F*_IS_, fixation index; DC, number of distance classes; 1st DC, maximum distance of the first class (m); *F*_ij_-intra, intra individual kinship coefficient; *F*_ij(1),_ average kinship coefficient of the first distance class; *Sp,* intensity of FSGS and *P*-value of one-sided test of the regression slope *b*; *b* mean jacknife ± SE, jackknife mean of *b* and standard error. ns, not significant; ***, *P*≤0.001; **, *P*≤0.01; *, *P*≤0.05.

| Population | n | SSR | *A* | *A*_R_(SD) | *H*_E_ | *F*_IS_ | DC | 1st DC (m) | *F_ij_-intra* | *F_ij(1)_* | *Sp* | *b* mean jackknife±SE |
| --- | --- | --- | --- | --- | --- | --- | --- | --- | --- | --- | --- | --- |
| BCI | 147 | 3 | 5.84 | 8.09 (2.60) | 0.808 | 0.157^***^ | 7 | 113 | 0.1538 | 0.0373 | 0.0223^***^ | -0.0215±0.0104 |
| Yasuní | 34 | 3 | 4.81 | 8.10 (2.69) | 0.727 | 0.054^ns^ | 10 | 94 | 0.1042 | -0.0634 | 0.0035^ns^ | -0.0038±0.0094 |
| Paracou | 148 | 3 | 9.99 | 12.57 (3.56) | 0.880 | 0.172^***^ | 4 | 203 | 0.1709 | 0.0154 | 0.0090^***^ | -0.0088±0.0029 |
| Ituberá | 85 | 3 | 3.53 | 5.26 (3.54) | 0.588 | 0.159^***^ | 5 | 151 | 0.1564 | 0.0082 | 0.0072^*^ | -0.0072±0.0045 |
| São Tomé | 42 | 3 | 4.69 | 8.98 (2.96) | 0.769 | 0.178^***^ | 6 | 856 | 0.1783 | 0.1135 | 0.0468^***^ | -0.0415±0.0166 |
| Nkong Mekak | 70 | 3 | 6.20 | 7.22 (5.94) | 0.729 | 0.205^***^ | 5 | 312 | 0.2051 | 0.0272 | 0.0169^***^ | -0.0165±0.0052 |
| Mbikiliki | 94 | 3 | 4.71 | 7.13 (3.96) | 0.686 | 0.265^***^ | 7 | 240 | 0.2650 | 0.0989 | 0.0372^***^ | -0.0335±0.0196 |

Table S4.2. Genetic diversity and fine-scale spatial genetic structure statistics in *Symphonia globulifera* based a) on 18 genic nuclear SSRs, data from Olsson *et al.* [2], b). on 3-5 genic nuclear SSRs and subsets of ca. 30 individuals. n, sample size for SSR data; *A*, mean number of alleles per locus; *A*_R_, allelic richness or number of alleles expected in a sample of 30 individuals and standard deviation; *H*_E_, expected heterozygosity; *F*_IS_, fixation index; DC, number of distance classes; 1st DC, maximum distance of the first class (m); *F*_ij_-intra, intra individual kinship coefficient; *F*_ij(1),_ average kinship coefficient of the first distance class; *Sp,* intensity of FSGS and *P*-value of one-sided test of the regression slope *b*; *b* mean jacknife ± SE, jackknife mean of *b* and standard error. ns, not significant; ***, *P*≤0.001; **, *P*≤0.01; *, *P*≤0.05, ., *P*≤0.1,.

| **a**  Population | n | SSR | *A* | *A*_R_(SD) | *H*_E_ | *F*_IS_ | DC | 1st DC (m) | *F_ij_-intra* | *F_ij(1)_* | *Sp* | *b* mean jackknife±SE |
| --- | --- | --- | --- | --- | --- | --- | --- | --- | --- | --- | --- | --- |
| Paracou | 32 | 18 | 4.89 | 2.77(2.06) | 0.491 | 0.102^*^ | 4 | 378 | 0.1015 | -0.0072 | 0.0018. | -0.0018±0.0012 |
| Ituberá | 31 | 18 | 2.94 | 2.59(0.96) | 0.370 | -0.291^***^ | 4 | 178 | -0.2828 | -0.0025 | -0.0021^ns^ | 0.0021±0.0042 |
| São Tomé | 30 | 18 | 3.24 | 2.99(2.35) | 0.341 | 0.123^**^ | 4 | 1371 | 0.0841 | 0.0470 | 0.0339^***^ | -0.0323±0.0081 |
| Nkong Mekak | 31 | 18 | 3.83 | 3.29(1.47) | 0.457 | -0.167^***^ | 7 | 204 | -0.1721 | 0.0106 | 0.0072. | -0.0071±0.0057 |

| **b** Population | n | SSR | *A* | *A*_R_(SD) | *H*_E_ | *F*_IS_ | DC | 1st DC (m) | *F_ij_-intra* | *F_ij(1)_* | *Sp* | *b* mean jackknife±SE |
| --- | --- | --- | --- | --- | --- | --- | --- | --- | --- | --- | --- | --- |
| Paracou | 32 (subset different from Olsson et al. 2016) | 3 | 14.67 | 11.28(4.42) | 0.865 | 0.169^***^ | 4 | 203 | 0.1676 | -0.0022 | 0.0067. | -0.0064±0.0065 |
| Ituberá | 31 (same as Olsson et al. 2016) | 5 | 8.8 | 6.98(5.23) | 0.622 | 0.108^**^ | 4 | 178 | 0.1051 | 0.0070 | 0.0055^ns^ | -0.0049±0.0031 |
| São Tomé | 30 (same as Olsson et al. 2016) | 5 | 11.6 | 9.42(3.06) | 0.824 | 0.211^***^ | 4 | 1371 | 0.2137 | 0.0707 | 0.0475^***^ | -0.0438±0.0139 |
| Nkong Mekak | 31 (same as Olsson et al. 2016) | 5 | 11.4 | 9.24(5.46) | 0.807 | 0.163^***^ | 7 | 204 | 0.1631 | 0.0136 | 0.0088. | -0.0088±0.0042 |

**Literature cited**

1. Degen B, Bandou E, Caron H. Limited pollen dispersal and biparental inbreeding in *Symphonia globulifera* in French Guiana. Heredity. 2004;93: 585–591. doi:10.1038/sj.hdy.6800560

2. Olsson S, Seoane Zonjic P, Bautista R, Claros MG, González-Martínez SC, Scotti I, et al. Development of genomic tools in a widespread tropical tree, *Symphonia globulifera* L.f.: a new low-coverage draft genome, SNP and SSR markers. Mol Ecol Resour. 2016; doi:10.1111/1755-0998.12605
