# Supplementary material for: Altitudinal gradients, biogeographic history and microhabitat adaptation affect fine-scale spatial genetic structure in African and Neotropical populations of an ancient tropical tree species
Source: PLoS One. 2017 Aug 3;12(8):e0182515. doi: 10.1371/journal.pone.0182515 (PMC5542443; doi:10.1371/journal.pone.0182515)
Supplement: S1 Table — (DOCX) [file pone.0182515.s005.docx]

**S1 Table. Estimates of mating system and FSGS parameters in genetic clusters of *Symphonia globulifera*.** GP, gene pool (GPs include individuals with ancestry proportions *Q* of 0.875-1); n, sample size; *F*_IS_, fixation index; *F*_IS_*, fixation index after null allele correction; DC, number of distance classes; 1^st^ DC, maximum distance of the first class (m); *Sp,* intensity of SGS and *P*-value of one-sided test of the regression slope *b*. ns, not significant; ***, *P*≤0.001; **, *P*≤0.01; *, *P*≤0.05; nc, not calculated (no null alleles or small sample size).

| Population | GP | n | *F*_IS_ | *F_IS_** | DC | 1^st^ DC (m) | *Sp* |
| --- | --- | --- | --- | --- | --- | --- | --- |
| São Tomé | GP 1 | 4 | nc | nc | - | - | - |
|  | GP 2 | 12 | 0.054^ns^ | nc | 3 | 1434 | -0.0008^ns^ |
|  | GP 3 | 8 | -0.025^ns^ | nc | 3 | 724 | -0.0054^ns^ |
| Nkong Mekak | GP 1 | 35 | 0.055^ns^ | nc | 7 | 353 | 0.0181^***^ |
|  | GP 2 | 14 | 0.119^*^ | nc | 3 | 219 | 0.0085^ns^ |
| Mbikiliki | GP 1 | 18 | -0.011^ns^ | nc | 3 | 125 | 0.0136^**^ |
|  | GP 2 | 45 | 0.125^***^ | 0.035 ^ns^ | 4 | 265 | 0.0136^***^ |
