# Supplementary material for: Altitudinal gradients, biogeographic history and microhabitat adaptation affect fine-scale spatial genetic structure in African and Neotropical populations of an ancient tropical tree species
Source: PLoS One. 2017 Aug 3;12(8):e0182515. doi: 10.1371/journal.pone.0182515 (PMC5542443; doi:10.1371/journal.pone.0182515)
Supplement: S2 Table — (DOCX) [file pone.0182515.s006.docx]

**S2 Table Altitudinal clustering of gene pools in *Symphonia globulifera* populations.** n, sample size range per altitudinal class; GP, gene pool; *P*-value, *P-*value of an ANOVA contrasting individual ancestry values (*q*) in three altitudinal classes for each GP within populations; *q* mean, mean *q* of individuals in altitude class H1, H2, or H3 (from lowest to highest). ns, not significant; ***, *P*≤0.001; **, *P*≤0.01; *, *P*≤0.05.

| Populations | n | GP | *P*-value | *q* mean (H1) | *q* mean (H2) | *q* mean (H3) |
| --- | --- | --- | --- | --- | --- | --- |
| BCI | 46-51 | GP 1 | ** | 0.310 | 0.245 | 0.422 |
|  |  | GP 2 | * | 0.389 | 0.341 | 0.282 |
|  |  | GP 3 | * | 0.301 | 0.414 | 0.296 |
| São Tomé | 12-16 | GP 1 | ns | 0.311 | 0.365 | 0.140 |
|  |  | GP 2 | *** | 0.073 | 0.347 | 0.831 |
|  |  | GP 3 | *** | 0.616 | 0.288 | 0.029 |
| Nkong Mekak | 23-24 | GP 1 | * | 0.693 | 0.461 | 0.739 |
|  |  | GP 2 | * | 0.307 | 0.539 | 0.261 |
| Mbikiliki | 31-32 | GP 1 | *** | 0.177 | 0.589 | 0.334 |
|  |  | GP 2 | *** | 0.823 | 0.411 | 0.666 |
